# Nutrient Control of Yeast Gametogenesis Is Mediated by TORC1, PKA and Energy Availability

**DOI:** 10.1371/journal.pgen.1006075

**Published:** 2016-06-06

**Authors:** Hilla Weidberg, Fabien Moretto, Gianpiero Spedale, Angelika Amon, Folkert J. van Werven

**Affiliations:** 1 David H. Koch Institute for Integrative Cancer Research and Howard Hughes Medical Institute, Massachusetts Institute of Technology, Cambridge, Massachusetts, United States of America; 2 Cell Fate and Gene Regulation Laboratory, The Francis Crick Institute, London, United Kingdom; National Cancer Institute, UNITED STATES

## Abstract

Cell fate choices are tightly controlled by the interplay between intrinsic and extrinsic signals, and gene regulatory networks. In *Saccharomyces cerevisiae*, the decision to enter into gametogenesis or sporulation is dictated by mating type and nutrient availability. These signals regulate the expression of the master regulator of gametogenesis, *IME1*. Here we describe how nutrients control *IME1* expression. We find that protein kinase A (PKA) and target of rapamycin complex I (TORC1) signalling mediate nutrient regulation of *IME1* expression. Inhibiting both pathways is sufficient to induce *IME1* expression and complete sporulation in nutrient-rich conditions. Our ability to induce sporulation under nutrient rich conditions allowed us to show that respiration and fermentation are interchangeable energy sources for *IME1* transcription. Furthermore, we find that TORC1 can both promote and inhibit gametogenesis. Down-regulation of TORC1 is required to activate *IME1*. However, complete inactivation of TORC1 inhibits *IME1* induction, indicating that an intermediate level of TORC1 signalling is required for entry into sporulation. Finally, we show that the transcriptional repressor Tup1 binds and represses the *IME1* promoter when nutrients are ample, but is released from the *IME1* promoter when both PKA and TORC1 are inhibited. Collectively our data demonstrate that nutrient control of entry into sporulation is mediated by a combination of energy availability, TORC1 and PKA activities that converge on the *IME1* promoter.

## Author Summary

The cell-fate controlling gametogenesis is essential for all sexual reproducing organisms. In *Saccharomyces cerevisiae*, entry into gametogenesis or sporulation is dictated by mating type and nutrient availability. These signals regulate the expression of the master regulator of entry into sporulation, *IME1*. In this manuscript we describe how nutrients control *IME1*. We show that inhibiting two highly conserved nutrient sensing pathways (PKA and TORC1) mimics starvation-induced sporulation and drives cells to induce *IME1* and complete meiosis in nutrient-rich conditions. In addition, we show that respiration and fermentation are interchangeable energy providers for entry into gametogenesis. Finally, we have uncovered a critical role for TORC1 during entry into gametogenesis. In addition to the known role of TORC1 in repressing *IME1*, we find that intermediate TORC1 activity is required for entry in gametogenesis. Too much or too little TORC1 activity inhibits gametogenesis. Our data explains how two conserved signalling pathways control a developmental decision essential for sexual reproduction, about which remarkably little is known in all eukaryotes. Thus the activities of two nutrient sensing pathways and energy availability determine whether cells enter gametogenesis or not.

## Introduction

Cellular differentiation programs are controlled by environmental and cell intrinsic events. How cells integrate multiple stimuli to regulate cell fate choice is poorly understood. The yeast *Saccharomyces cerevisiae* is an ideal model to study this problem. In response to multiple, well-defined signals, yeast cells induce a differentiation program to form four haploid gametes or spores [1, 2]. Gametogenesis or sporulation is characterized by a specialized cell division called meiosis. During sporulation diploid cells undergo a single round of DNA replication followed by two consecutive nuclear divisions, meiosis, to generate progeny containing half the number of chromosomes of the diploid parent cell.

The initiation of gametogenesis is controlled by cell-intrinsic and cell-extrinsic signals, which together regulate a single master transcription factor called inducer of meiosis I, *IME1* [3, 4]. In cells expressing a single mating type, *MAT*a or *MAT*α, *IME1* is repressed by transcription coupled repression of the *IME1* promoter involving the long noncoding RNA *IRT1* [5]. In *MAT*a/α diploid cells Rme1, the transcriptional activator of *IRT1*, is repressed. As a consequence these cells express *IME1* upon nutrient deprivation [6]. For efficient *IME1* induction a fermentable carbon source and nitrogen need to be absent from the growth medium. Under these conditions cells produce ATP via oxidative phosphorylation to facilitate *IME1* expression [7, 8].

Two conserved signalling pathways have been implicated in nutrient regulation of *IME1* expression. First, the presence of glucose in the growth medium activates the Ras/cAMP-dependent Protein Kinase A (PKA) pathway, which in turn inhibits *IME1* and entry into sporulation [9, 10]. The second regulator of *IME1* is the target of rapamycin complex I (TORC1). TORC1 promotes macromolecule biosynthesis in response to nitrogen and amino acid availability [11]. When nitrogen sources/amino acids are ample, TORC1 is active and inhibits *IME1* and sporulation [7, 12]. Whether PKA and TORC1 are the main mediators of nutrient control of *IME1*, and how the two pathways control entry into sporulation is not well understood.

Here we describe how nutrients control *IME1* expression. We find that PKA and TORC1 signalling account for the majority of *IME1* regulation by nutrients. Inhibition of PKA and TORC1 activity is sufficient to induce *IME1* expression even in the presence of high levels of nutrients. Under these conditions, cells induce *IME1*, complete meiosis, and generate spores with kinetics that are highly reminiscent of those observed under starvation conditions. The ability to induce sporulation in the presence of ample nutrients further allowed us to investigate the importance of respiration and TORC1 activity for the induction of gametogenesis. We find that respiration and fermentation are interchangeable for *IME1* induction. Both metabolic pathways can serve as energy providers during entry into sporulation. Our analysis further shows that intermediate levels of TORC1 activity are critical for gametogenesis. When TORC1 is fully active or completely inhibited, *IME1* is repressed. Finally, we show that the transcriptional repressor Tup1 binds to and represses the *IME1* promoter when TORC1 and/or PKA are active, but not when both pathways are inhibited. Importantly, depletion of Tup1 is sufficient to mimic starvation-induced *IME1* expression. Our data demonstrate that nutrient control of sporulation is sensed and orchestrated by TORC1 and PKA signalling pathways and by the availability of energy.

## Results

### Inhibition of PKA and TORC1 in nutrient rich medium mimics starvation induced *IME1* expression

**Fig 1:**
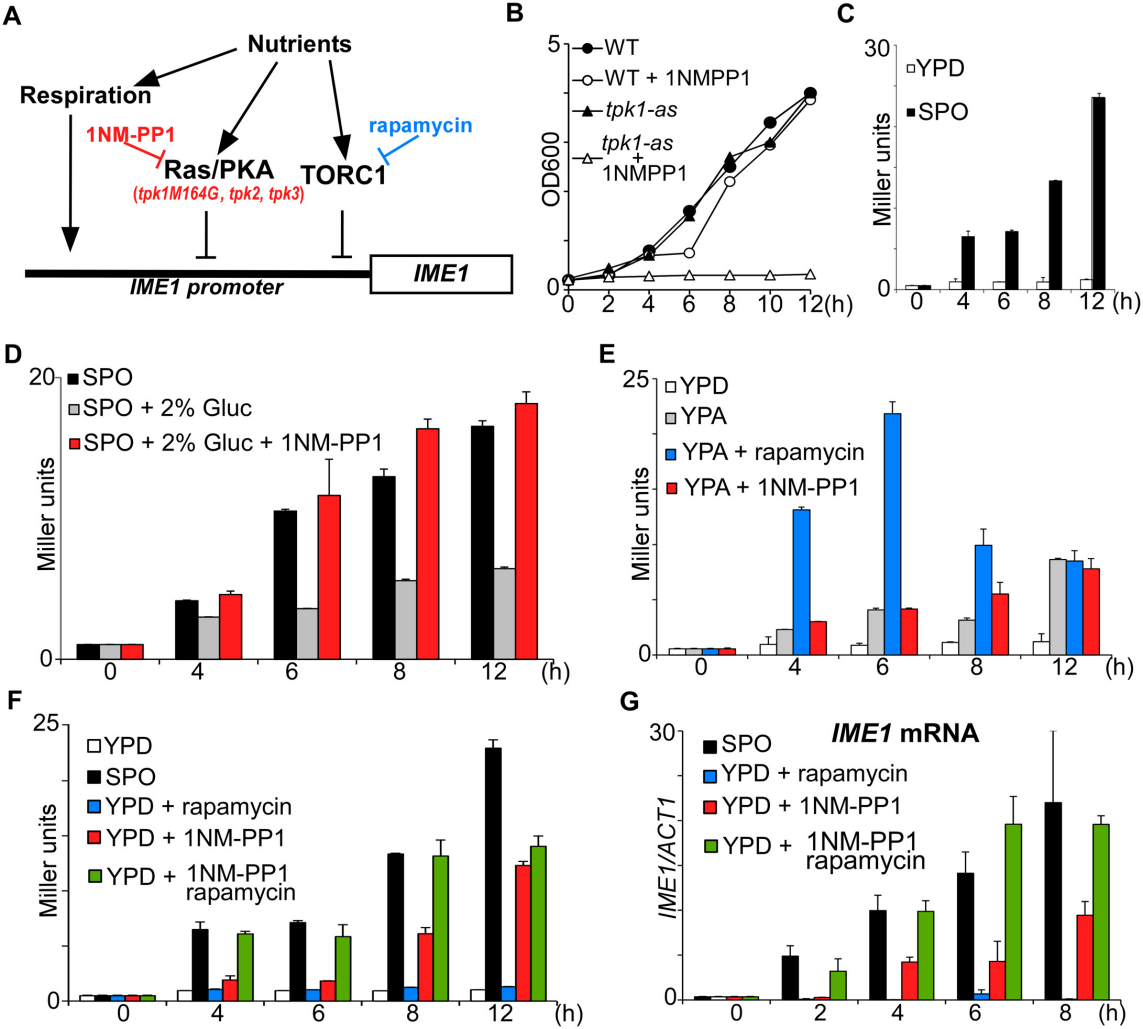
Inhibition of PKA and TORC1 induces *IME1* and meiosis in rich medium. (A) Scheme of the signalling pathways controlling *IME1* expression. 1NM-PP1 and rapamycin compounds inhibit *tpk1-as* and TORC1 respectively. (B) Wild-type (FW1511) cells or cells harbouring the *tpk1M164G*, *tpk2*Δ, *tpk3*Δ alleles (*tpk1-as*, FW1762) were grown in YPD overnight, diluted to 0.2 (OD600), and subsequently cells were treated with 5μM 1NM-PP1or untreated. Cell density (OD600) was measured over time at the indicated time points. (C) *IME1* promoter activity was measured in a diploid *tpk1-as* strains and the *IME1* promoter fused to *LacZ* reporter (FW1976). Cells were grown in YPD overnight, diluted into YPD or shifted to sporulation medium (SPO), and samples were taken after 0, 4, 6, 8, 10, and 12 hours. β-galactosidase activity was measured using a quantitative liquid ortho-nitrophenyl-β-galactoside (ONPG) assay (see Materials and Methods for details). The promoter activities are displayed in Miller Units, and the standard error of the mean of at least two biological experiments is shown. (D) Similar to C except that cells were shifted to SPO, SPO plus 2% glucose or SPO with 2% glucose and 5μM 1NM-PP1. Samples were taken at the indicated time points. (E) Similar to C, except that samples shifted to YPD or YP-acetate (YPA), YPA plus rapamycin or YPA plus 1NM-PP1. (F) Similar to C except that cells were shifted to SPO or YPD and treated with rapamycin, 1NM-PP1 or both compounds. (G) *IME1* mRNA quantification of cells shifted from YPD to SPO or YPD plus rapamycin, 1NM-PP1, or both compounds. Samples were taken at the indicated time points. Total RNA was isolated, reverse transcribed, and *IME1* mRNA levels were measured by quantitative PCR. Signals were normalized to *ACT1* levels. The standard error of the mean of at least two biological experiments is shown.

In budding yeast nutrient availability determines whether cells enter sporulation. The PKA and TORC1 pathways as well as respiration have been linked to the regulation of *IME1* expression by nutrients and to entry into sporulation (Fig 1A) [1]. To determine whether TORC1 and PKA are the major mediators of nutrient sensing in triggering sporulation, we examined how inactivation of either or both pathways affects *IME1* expression. TORC1 can be rapidly and efficiently inhibited using the small molecule rapamycin that reduces cell proliferation rate significantly (S1A Fig). Inhibition of the PKA pathway is more complex because budding yeast encodes three redundant genes encoding the catalytic subunits of PKA, *TPK1*, *TPK2*, and *TPK3* [13]. To inhibit the PKA pathway, we generated an ATP analogue sensitive strain of PKA that we define as *tpk1-as*. The strain contains gene deletions in *TPK2*, *TPK3* and a point mutation in *TPK1* (*tpk1M164G*) that transforms this allele into an ATP analog sensitive (as) allele [14]. In the *tpk1-as* strain, PKA activity can be specifically blocked using the ATP analog 1NM-PP1, which results in a growth arrest (Fig 1B and S1A Fig).

To measure *IME1* promoter activity in response to modulating PKA activity, we used an *IME1*-promoter LacZ reporter fusion (*pIME1-LacZ*) that was integrated at the *IME1* locus without disrupting the endogenous *IME1*. This fusion serves as an accurate readout for *IME1* promoter activity [7]. When we shifted control and *tpk1-as* diploid cells from rich medium containing glucose (YPD) to sporulation medium (SPO), a condition which induces *IME1*, β-galactosidase activity increased (S1B Fig and Fig 1C). The β-galactosidase levels were comparable between the two strains suggesting that *tpk1-as* allele does affect *IME1* regulation in SPO (S1B Fig). As expected, *IME1* promoter activity did not increase when *tpk1-as* cells were shifted to fresh YPD (Fig 1C).

Using the *tpk1-as* and *pIME1-LacZ* system, we first determined whether glucose repression of *IME1* is mediated by PKA signalling. Cells were shifted from YPD to SPO, or to SPO medium containing glucose in the presence or absence of the ATP analog 1NM-PP1 (Fig 1D). *IME1* promoter activity was strongly reduced in the presence of glucose. In contrast, when PKA was inhibited *IME1* promoter activity was comparable with cells grown in the absence of glucose. This result shows that glucose inhibits *IME1* expression predominantly via the PKA signalling pathway.

The presence of a nitrogen source also prevents *IME1* expression [3]. To test whether TORC1 signalling is responsible for *IME1* repression by nitrogen sources and amino acids we examined the effects of rapamycin on *IME1* expression. To exclude the effects of glucose repression on *IME1*, we used a nitrogen and amino acid rich medium containing the non-fermentable carbon source acetate (YPA) but lacking a fermentable carbon source. We found that *IME1* promoter activity slightly increased in cells shifted from YPD to YPA, and, inhibition of PKA did not further increase *IME1* expression (Fig 1E and S1D Fig). This is expected because it is known that Ras/PKA transmits the glucose signal and thus glucose levels control *IME1* via PKA [3, 9, 10, 15, 16]. When we inhibited TORC1, by treating cells grown in YPA medium with rapamycin, *IME1* was rapidly induced. The majority of cells (95%) were also able to form spores within 24 hours (S1 Table). We conclude, as reported previously, that the PKA pathway transmits the glucose signal to the *IME1* promoter, and that TORC1 most likely transmits the nitrogen signal [3, 9].

To examine whether PKA and TORC1 are the major mediators of nutrient control of *IME1* expression, we inhibited either or both pathways in cells grown in rich medium containing glucose (YPD) (Fig 1F and S1C Fig). Inhibition of TORC1 had no effect on *IME1* expression. In contrast, *IME1* promoter activity strongly increased between 8 to 12 hours following treatment with PKA inhibitors. Interestingly, when both PKA and TORC1 were inhibited, *IME1* induction was already noticeable at 4 hours, and peaked at 8 hours and was remarkably similar to levels seen in cells incubated in SPO medium (Fig 1F and S1 Fig). Similar results were obtained when *IME1* mRNA levels were examined (Fig 1G). These data show that the combined inhibition of PKA and TORC1 activities is sufficient to mimic nutrient control of *IME1* expression. We conclude that TORC1 and PKA are two major mediators of nutrient regulation of *IME1* expression.

### Inhibition of PKA and TORC1 induces *IME1* expression in the majority of cells

**Fig 2:**
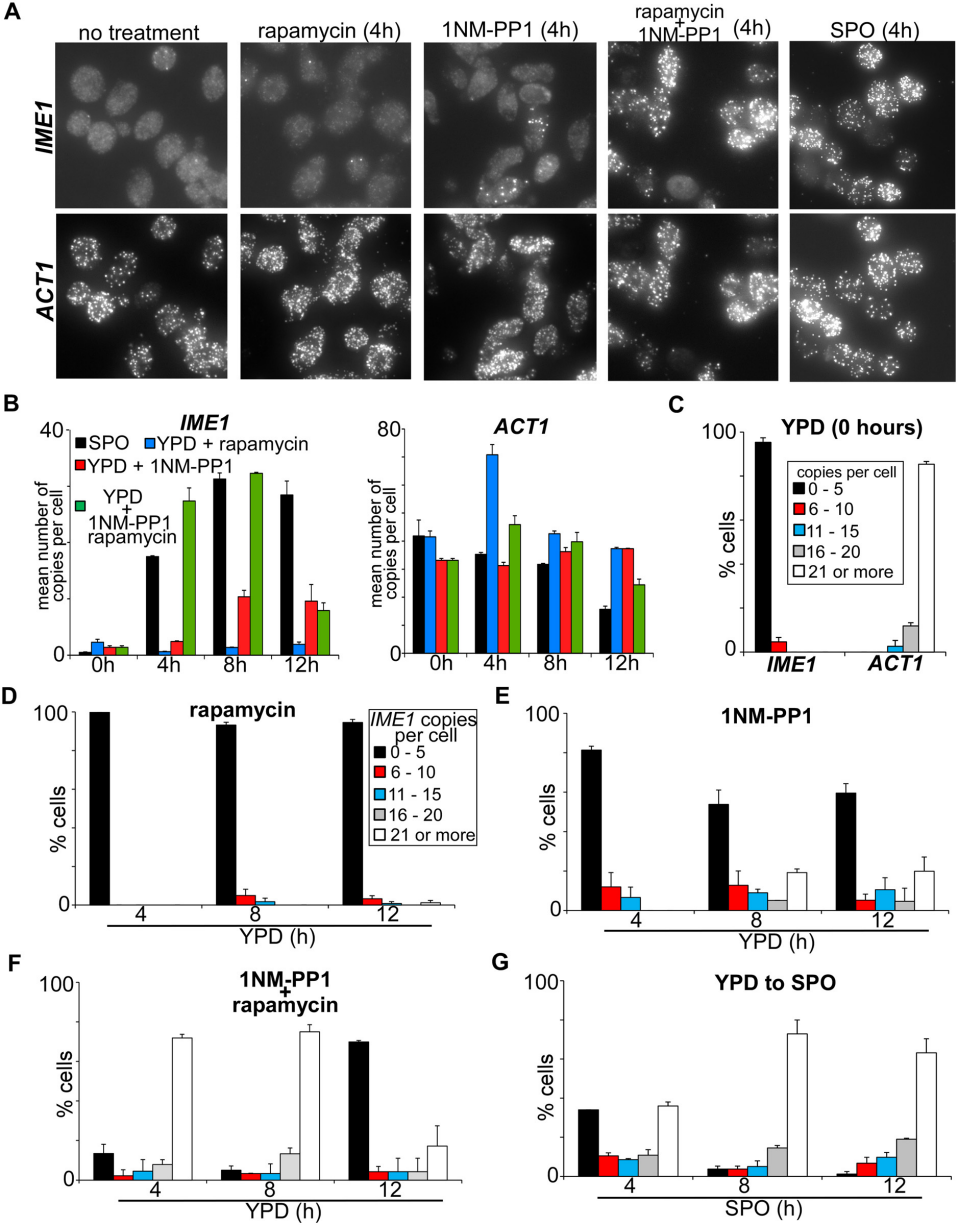
Single cell quantification of *IME1*. (A) Representative images used for the analyses of *IME1* and *ACT1* transcript levels in single cells. Cells harbouring *tpk1-as* (FW1762) were grown in YPD overnight and were either shifted to SPO, or diluted into fresh YPD plus rapamycin, 1NM-PP1 or 1NM-PP1/rapamycin. Cells were fixed, hybridized with probes directed against *IME1* (AF594) and *ACT1* (Cy5), and were imaged (see Materials and Methods for details). *ACT1* was used as an internal control and only *ACT1* positive cells were selected for the analysis. (B) Mean of *IME1* and *ACT1* transcripts’ number among single cells as described in A. Cells harbouring *tpk1-as* (FW1762) were grown in YPD overnight, diluted into fresh YPD plus 1NM-PP1 or 1NM-PP1/ rapamycin and samples were taken at the indicated time points. Cells were imaged and quantified (see Materials and Methods for details). At least, 60 cells (n = 60) were quantified per time point. The standard error of the mean of at least two biological experiments is shown. (C) Distribution of *IME1* and *ACT1* transcripts among single cells grown in YPD as described in A and B. Cells were binned in five classes of transcript levels (0 to 5; 6 to 10; 11 to 15; 16 to 20 and 21 or more transcripts per cell) and the percentages of each class contributing to the total population are indicated. At least, 60 cells (n = 60) were quantified per time point. The standard error of the mean of at least two biological experiments is shown. (D-G) Similar to C except that cells were grown in YPD overnight and transferred to YPD plus rapamycin (D), YPD plus 1NM-PP1 (E), YPD plus 1NMPP and rapamycin (F), or SPO (G). Samples were taken at the indicated time points. Cells were binned in five classes of transcript levels (0 to 5; 6 to 10; 11 to 15; 16 to 20 and 21 or more transcripts per cell) and the percentages of each class contributing to the total population are indicated.

Our results show that inhibition of PKA leads to some degree of *IME1* expression in rich medium (YPD) (approximately 50% of that observed in SPO medium; Fig 1F and 1G). One explanation for this observation is that PKA inhibition induces *IME1* at low or intermediate levels in all cells. It is also possible that *IME1* induction occurs only in a subpopulation of cells when PKA is inhibited. To distinguish between these possibilities, we measured the distribution of *IME1* expression in cells by single molecule RNA fluorescence *in situ* hybridization (smFISH) (Fig 2A). The technique can reliably measure absolute transcript levels in single cells [17]. To ensure that the signals were specific and probes entered the cells, we first measured *IME1* and *ACT1* transcript levels in wild-type and *ime1*Δ diploid cells that were induced in SPO medium (S2 Fig). While *IME1* was expressed in the wild type, no transcripts were detected in *ime1*Δ cells. As expected, *ACT1* levels were comparable between the two strains (S2B Fig). Next, we counted the mean number of *IME1* transcripts in cells grown in rich medium shifted to SPO medium, or treated with PKA or PKA/TORC1 inhibitors (Figs 2A and 1B). The *IME1* expression pattern matched the RT-PCR experiment (compare Figs 1G and 2B). In cells treated with the PKA inhibitor *IME1* levels increased after 8 hours to about 10 copies per cell on average. When both TORC1 and PKA were inhibited, *IME1* was transcribed efficiently and cells contained on average 30 copies per cell at 8 hours after treatment, which was comparable to *IME1* levels in SPO medium (Fig 2B).

It is worth noting that *IME1* mRNA levels decline sharply 12 hours after inhibition of the PKA and TORC1 pathways but remained elevated in cells incubated in SPO medium for the same amount of time (Fig 2B). Given that expression of *IME1* is known to decline when cells undergo meiotic divisions, a plausible explanation is that progression into meiosis differs between the two conditions [18]. Indeed, when PKA and TORC1 were inhibited the majority of cells underwent meiotic divisions within 12 hours (see next section for details). In contrast, when cells were directly transferred from YPD medium into SPO medium sporulation did not occur efficiently, and it is likely that many (more than 50%) of the cells were arrested in intermediate stages of meiosis (S1 Table). Therefore a decline in *IME1* was not observed at 12 hours (Fig 2B). Finally, we would like to point out that we did not see a decline in *pIME1*-*LacZ* reporter activity even when cells progressed into meiosis (Fig 1F). This can be explained by the long half-life of β-galactosidase (more than 20 hours) [19].

Next, we analysed *IME1* abundance in single cells. We binned the single cell expression data into five classes of transcript levels (0–5, 6–10, 11–15, 16–20 and 21 or more transcripts). In untreated cells more than 95 percent of cells had no or low levels of *IME1*, whereas *ACT1* was expressed strongly in the majority cells (Fig 2C). In line with previous observations, rapamycin treatment had no effect on *IME1* expression (Fig 2D). Interestingly, when PKA was inhibited the majority of cells expressed no or low levels of *IME1* (0–5 copies per cell), but approximately 20% of cells expressed high levels of *IME1* (21 or more copies per cell) (Fig 2E). When both PKA and TORC1 were inhibited the majority of cells (more than 75%) harboured high levels of *IME1* RNA, which was comparable to SPO medium (compare Fig 2F and 2G). These data complement our population based assays and show that inhibition of PKA/TORC1 leads to significant *IME1* expression in the majority of cells grown in nutrient-rich conditions.

### Inhibition of TORC1/PKA induces meiosis

**Fig 3:**
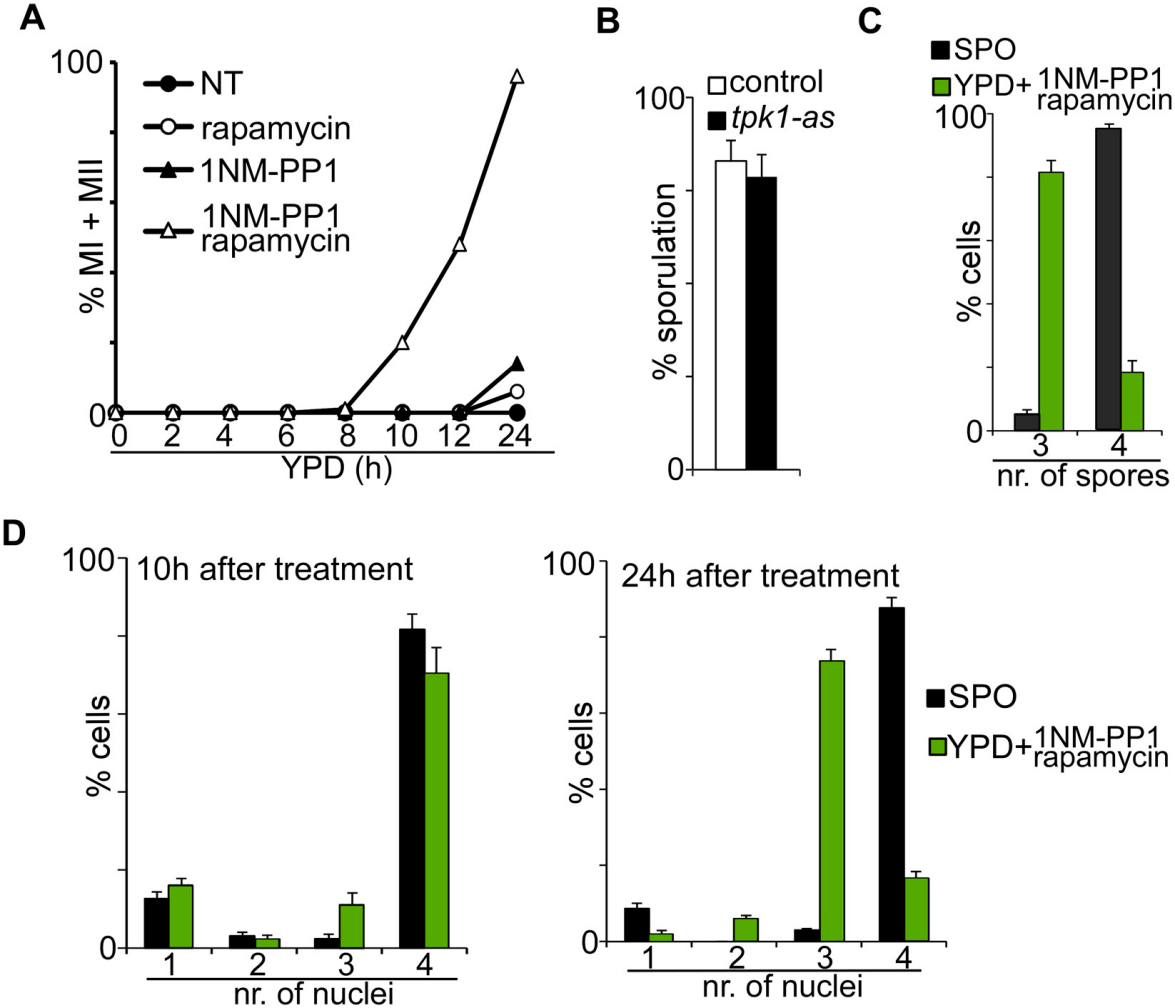
Inhibition of PKA and TORC1 induces meiosis. (A) Kinetics of meiotic divisions (MI + MII) of cells harbouring the *tpk1-as* allele (FW1762) shifted from YPD to fresh YPD with no treatment (NT) (closed circles), rapamycin (open circles), 1NM-PP1 (closed triangles), or rapamycin/1NM-PP1 (open triangles). Samples were taken at the indicated time point, fixed, and DAPI masses were counted. Cells that contain 2, 3, or 4 DAPI masses were classified as cells that underwent meiosis. For each time point 100 cells were counted. (B) Quantification of sporulation efficiencies in control (FW1511) and *tpk1-as* (FW1762) diploid cells. Cell were grown overnight in YPD, shifted to pre-sporulation medium, and then transferred to sporulation medium (SPO). Percentages of sporulated cells were determined after 48 hours in SPO. (C-D) Quantification of number of nuclei and spores. Cells harbouring the *tpk1-as* allele were grown in YPD, shifted to pre-sporulation medium, and subsequently shifted to SPO medium or to YPD plus 1NM-PP1 and rapamycin. The percentage of cells harbouring three or four spores was determined at 24 hours after treatment (C). DAPI labelled nuclei were counted at 10 hours (D, left panel) and 24 hours (D, right panel) after treatment. For each time point, 200 cells were counted.

Little is known about how nutrient signalling controls other aspects of sporulation. To determine the consequences of PKA and TORC1 inhibition on meiotic progression, we examined subsequent stages of meiosis by measuring the kinetics of meiosis in cells shifted to YPD containing inhibitors of PKA and TORC1. Interestingly, less than 15% of cells underwent meiotic divisions when only PKA was inhibited. Given that a significant higher portion of cells were positive for intermediate or high levels of *IME1* compared to number of cells that underwent meiotic divisions (Fig 2D and 2E), the result suggests that events downstream of *IME1* are perhaps not efficiently induced in these cells. More than 90% of cells underwent meiotic divisions when both PKA and TORC1 were inhibited (Fig 3A). Under this condition cells also formed spores, but 70 percent (224 out 320 spores) of spores formed colonies compared to 95 percent (304 out of 320 spores) for the wild-type cells induced to sporulate in SPO medium. This reduced spore viability was not due to the *tpk1-as* allele being hypomorphic, because the same strain sporulated efficiently and exhibited 94 percent (301 out of 320 spores) spore viability in SPO medium (Fig 3B). Apart from reduced spore viability, we also observed a strong enrichment for triads in cells treated with PKA/TORC1 inhibitors (Fig 3C). Although four DAPI masses formed during the two meiotic divisions, three were packaged into spores and one nucleus was evicted or degraded from cells (Fig 3C and 3D). We conclude that inhibition of TORC1 and PKA is sufficient to drive entry into and progression through the sporulation program.

### Fermentation and respiration are interchangeable energy providers for *IME1* transcription

Respiration is needed for *IME1* transcription and for entry into sporulation [7, 8]. However, it is not clear whether *IME1* expression is dependent on the energy produced by respiration or whether it requires a signal from active mitochondria. The system we developed to induce sporulation in the presence of ample nutrients allowed us to investigate this question.

**Fig 4:**
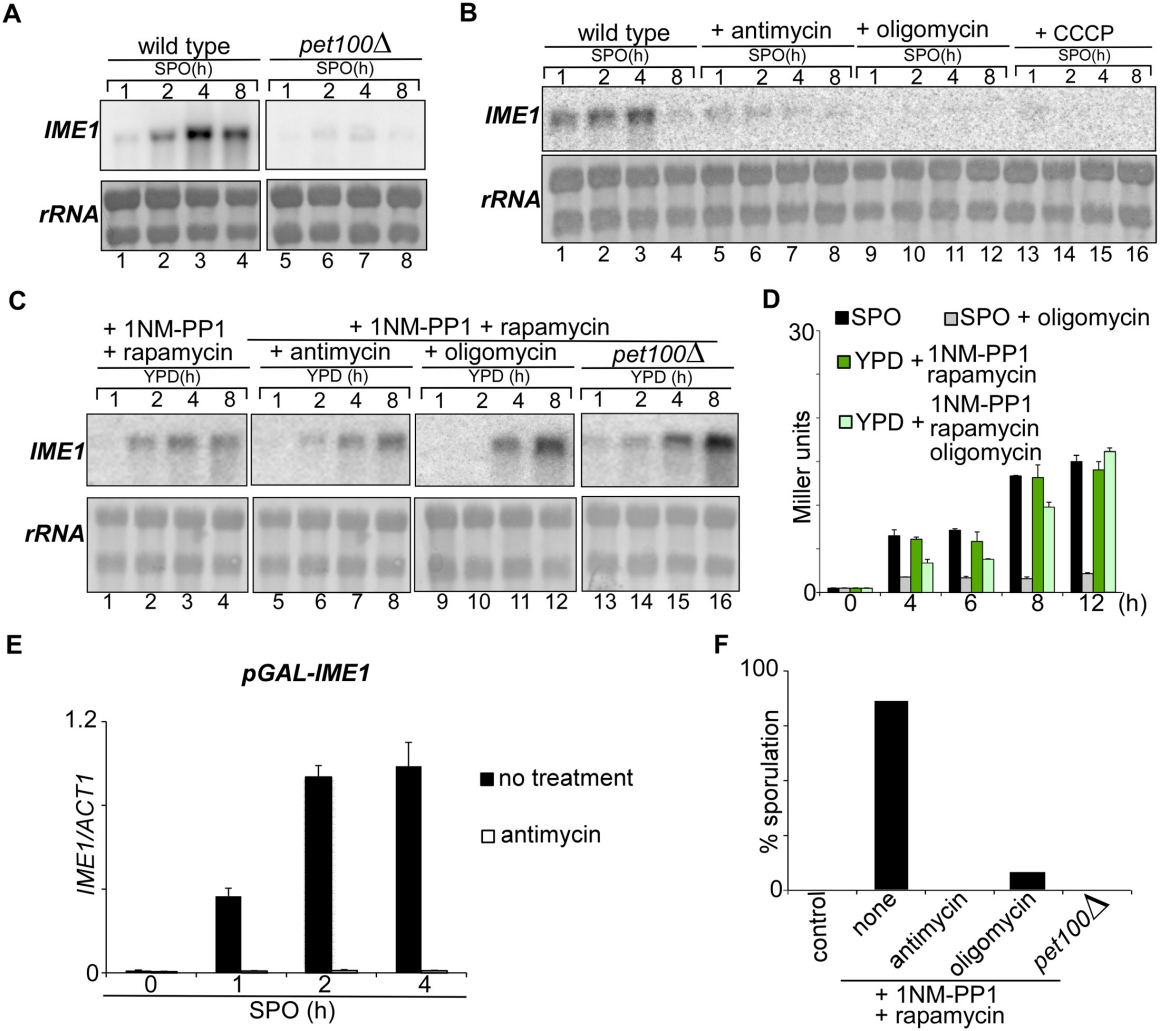
Respiration is not essential to *IME1* induction. (A) Northern blot analysis of *IME1* expression in control (FW1762, lanes 1–4) and *pet100*Δ mutant (FW1770, lanes 5–8). Cells were grown overnight in YPD medium and shifted to sporulation medium (SPO), and samples were taken at the indicated time points. (B) Similar to A except that cells (FW1762) were grown in pre-sporulation medium before shifted to SPO. Subsequently, cells were either not treated (lanes 1–4), treated with antimycin A (50 μM, lanes 5–8), oligomycin (10 μM, lanes 9–12), or with CCCP (20 μM, lanes 13–16). (C) Similar to A, except that cells were diluted into 1NM-PP1 and rapamycin containing YPD (lanes 1–16), and treated with antimycin A (lanes 5–8) or oligomycin (lanes 9–12). The *pet100*Δ mutant (lanes 13–16) was also included in the analyses. (D) *IME1* promoter activity was measured using a diploid strain harbouring *tpk1-as* and the *IME1* promoter fused to *LacZ* reporter (FW1976). Cells were grown in YPD overnight, diluted into YPD plus 1NM-PP1/rapamycin or shifted to sporulation medium (SPO) in the presence or absence of oligomycin (10 μM). Samples were taken after 0, 4, 6, 8, and 12 hours. β-galactosidase activity was measured using a quantitative liquid ortho-Nitrophenyl-β-galactoside (ONPG) assay (see Materials and Methods for details). The promoter activities are displayed in Miller Units, and the standard error of the mean of at least two biological experiments is shown. (E) Quantification of *IME1* mRNA levels in a strain harbouring the *GAL1* promoter fused to *IME1* and *GAL4-ER* (FW3243). Cells were grown in YPD, shifted to pre-sporulation medium, and transferred to SPO in the absence or presence of antimycin A. The *GAL1* promoter was activated using estradiol. Total RNA was isolated, reverse transcribed, and *IME1* mRNA levels were measured by quantitative PCR. Signals were normalized to *ACT1* levels. The standard error of the mean of at least two biological experiments is shown. (F) Quantification of sporulation efficiency of strains and treatments described in C. At least n = 200 cells were counted at 48 hours after treatment. Untreated diploid cells were included as a negative control.

First, we analysed how *IME1* expression is affected in respiratory deficient cells. Pet100 is required for the assembly of cytochrome c oxidase. Yeast cells lacking the *PET100* gene cannot respire. In line with previous observations, *pet100*Δ cells did not induce *IME1* in SPO medium (Fig 4A, compare lanes 1–4 to 5–8) [7]. Likewise, cells treated with the drugs antimycin A (antimycin), which inhibits cytochrome c reductase, or oligomycin, which inhibits the Fo subunit of the mitochondrial ATP synthase, did not induce *IME1* (Fig 4B, compare lanes 1–4 to 5–8 and 9–12). Uncoupling of respiration from energy production by treating cells with CCCP which disrupts the proton gradient and thus reduces the ability of the ATP synthase to function led to similar results (Fig 4B, compare lanes 1–4 and 13–16). Thus, respiration is required for induction of *IME1* expression in sporulation medium.

Next, we induced sporulation by inhibiting PKA and TORC1 in cells grown in glucose-rich medium, and tested whether respiration is required for *IME1* expression. To our surprise, *IME1* expression levels were comparable between control cells, and antimycin or oligomycin treated cells (Fig 4C, compare lanes 1–4 to 5–8 or 9–12). *pet100*Δ cells grown in YPD strongly induced *IME1* when the PKA and TORC1 pathways were inhibited (Fig 4C, compare lanes 1–4 to 13–16). To further quantify *IME1* promoter activity in respiratory deficient cells, we measured *pIME1-LacZ* reporter expression in oligomycin treated cells. As expected, in SPO medium the *IME1* promoter stayed repressed when cells were treated with oligomycin. In YPD plus TORC1 and PKA inhibitors, *IME1* expression accumulated with slightly slower kinetics in the presence of oligomycin but peaked to similar levels as control cells (Fig 4D). Finally we examined whether *IME1* can be induced from a heterologous promoter in SPO medium when respiration is inhibited (Fig 4E). When we induced *IME1* from the *GAL1* promoter using a Gal4-estrogen receptor fusion (*GAL4-ER)* that can be activated by the addition of estradiol, *IME1* was strongly induced. However, in cells treated with antimycin *IME1* stayed repressed. Previous work showed that expression of mRNAs from different genes is also affected under this condition [7]. We propose that the effects are not specific for *IME1*, but either transcription or mRNA stability or both are generally affected when cells are starved and cannot respire. Notably, even though cells were able to express *IME1* when respiration was inhibited in YPD medium with TORC1 and PKA inhibitors the vast majority of these cells did not complete gametogenesis (Fig 4F) indicating that other stages of sporulation require respiration. In conclusion, when sporulation is induced in the presence of ample nutrients, respiration is not required for *IME1* expression. This result suggests that either respiration or fermentation can serve as energy providers for induction of *IME1* transcription.

### TORC1 activity enables efficient *IME1* induction and meiosis

Our results show that inhibition of PKA and TORC1 activity is sufficient to initiate entry into sporulation. Although it is well established that PKA signalling inhibits sporulation, inhibition of TORC1 by rapamycin treatment has been reported to affect sporulation with different outcomes. We and others have shown that rapamycin can stimulate sporulation by inducing *IME1* expression [7]. Moreover, inactivation of TORC1 was shown to stabilize Ime1 and promotes its nuclear localization [20]. However, others have found that rapamycin can also inhibit spore formation in budding and fission yeast when added to the SPO medium [21, 22]. These seemingly conflicting results prompted us to further examine how rapamycin and TORC1 control sporulation.

**Fig 5:**
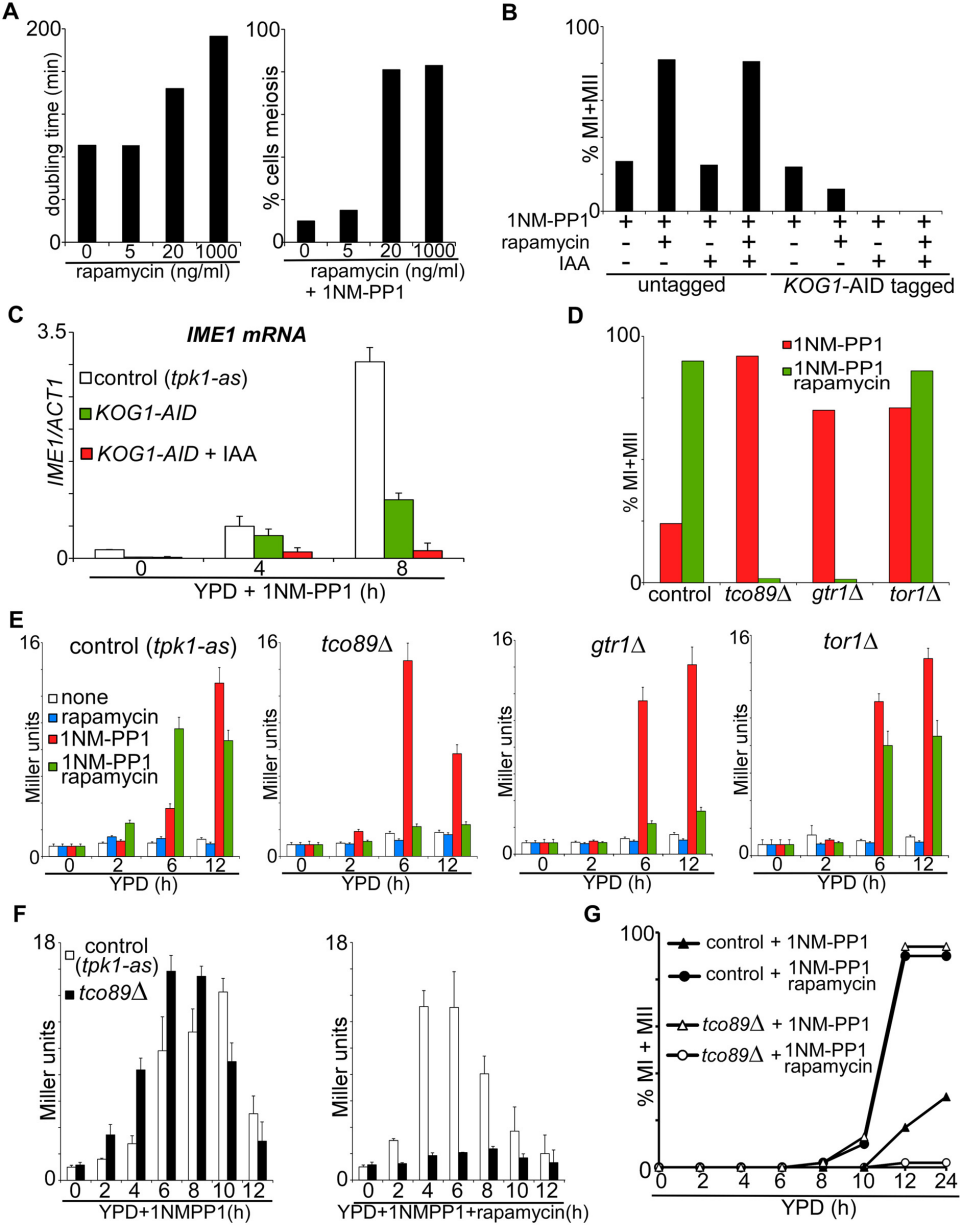
TORC1 activity is required for sporulation. (A) Cells (FW1762) were treated with different concentrations of rapamycin, and doubling times (left panel) as well as the fraction of cells that underwent meiosis (right panel) were quantified. Left panel, cells were grown in YPD, shifted to YPD plus 0, 5, 20, or 1000 ng/ml rapamycin and doubling times were measured during exponential growth. Right panel, cells were diluted into YPD plus PKA inhibitors and treated with different concentrations of rapamycin as indicated. DAPI masses were counted after 48 hours of treatment. (B) Control (FW1762) and *KOG-AID*/*pTEF1-osTIR1* (FW1904) cells harbouring *tpk1-as* were grown in YPD overnight, diluted into fresh YPD and treated with 1NM-PP1, rapamycin or IAA. The nuclei number in cells was counted after 48 hours of treatment by DAPI staining, and percentage of cells that underwent meiosis (MI+MII) was quantified. (C) Quantification of *IME1* mRNA levels in control (FW1762) and *KOG1-AID*/*pTEF1-osTIR1* (FW1904) cells harbouring *tpk1-as* and treated with 1NM-PP1. *KOG1-AID*/*pTEF1-osTIR1* cells were also treated with IAA. Samples were taken at the indicated time points. Total RNA was isolated, reverse transcribed, and *IME1* mRNA levels were measured by quantitative PCR. Signals were normalized to *ACT1* levels. The standard error of the mean of at least two biological experiments is shown. (D) Percentage of cells that underwent meiotic divisions (MI+MII) was determined in gene deletion strains, all harbouring *tpk1-as* and *pIME1-LacZ* (FW1976, control). The following gene deletion mutants were used for the analyses: control (FW1976), *tco89*Δ (FW2154), *gtr1*Δ (FW2164) or *tor1*Δ (FW2162). Samples were grown in YPD medium, fixed, and DAPI masses were counted at 48 hours after treatment with 1NM-PP1 or with 1NM-PP1 and rapamycin. (E) *IME1* promoter activity was measured in strains described in D. Cells were grown in YPD overnight, diluted into YPD plus 1NMPP1 and/or rapamycin, and samples were taken after 0, 4, 8, and 12 hours. β-galactosidase activity was measured using a quantitative liquid ortho-Nitrophenyl-β-galactoside (ONPG) assay (see Materials and Methods for details). The promoter activities are displayed in Miller Units, and the standard error of the mean of at least two biological experiments is shown. (F) *IME1* promoter activity was measured as described in E for control (FW1976) and *tco89*Δ (FW2154) strains. Cells were grown in YPD overnight, diluted into YPD plus 1NMPP1 and/or rapamycin, and samples were taken after 0, 2, 4, 6, 8, 10, 12 and 24 hours. (G) Kinetics of meiotic division (MI+MII) of strains and treatments described in F. Samples were taken at the indicated time points, fixed, and DAPI masses were counted.

First, we tested whether there is a concentration dependent effect of rapamycin on cell growth and sporulation. Rapamycin treatment at the concentration which ensures efficient sporulation (1000 ng/ml) diminished, but did not stop cell proliferation (190 min versus 90 min in control cells; Fig 5A). This observation suggests that TORC1 is still active. When we used 50 fold less rapamycin (20 ng/ml), cell proliferation was somewhat affected (145 min versus 90 min in control cells), and cells sporulated efficiently when combined with inhibition of PKA (Fig 5A, right panel). Lower concentrations of rapamycin had no observable effect on growth and sporulation. These results indicate that the TORC1 pathway is not completely blocked upon entry into sporulation and meiosis.

In line with our observation that rapamycin does not abolish growth completely, a recent study showed that rapamycin, irrespective of the concentration used, does not fully inhibit TORC1 activity [23]. Depletion or inactivation of the Kog1 subunit of the TORC1 complex, however, causes a complete growth arrest and a starvation response [24, 25]. We therefore depleted Kog1 using an auxin induced degradation system (AID) system [26] and examined the effects on *IME1* expression and sporulation. The system relies on *Oryza sativa TIR1* (*pTEF1-osTIR1*), which interacts with the SCF ubiquitin ligase, and the chemical indole-3-acetic acid (IAA), which allows for the SCF-TIR1 and E2 ubiquitin ligases to come together to polyubiquitinate and subsequently degrade AID by the proteasome [26]. Tagging Kog1 with the AID-tag decreased Kog1 activity as judged by reduced proliferation of cells carrying the *KOG1-AID* allele (S3A Fig). However, when *KOG1-AID* cells expressing *pTEF1-osTIR1* were treated with IAA to deplete Kog1, growth and proliferation were completely abolished (S3A and S3B Fig). The AID tag also partially interfered with sporulation as Kog1-AID cells exhibited reduced meiosis efficiency following treatment with PKA inhibitors and rapamycin (Fig 5B). Nonetheless, it was evident that depletion of Kog1 strongly affected *IME1* expression and as a result meiosis did not occur (Fig 5B and 5C). In conclusion, when we induce sporulation by inhibiting PKA in nutrient rich conditions, Kog1 is required for entry into sporulation.

Given that inactivation of TORC1 by depleting Kog1 abolished the cells’ ability to sporulate, we hypothesized that some TORC1 activity is needed for entry into sporulation. To test this hypothesis we modulated TORC1 activity. A number of TORC1 pathway mutants have been isolated previously and have been shown to reduce basal TORC1 activity [27–29]. Typically, these mutants are hypersensitive to rapamycin and some mutants cannot recover growth after rapamycin treatment. If reduced TORC1 activity is necessary for entry into sporulation, such mutants should sporulate in the presence of a nitrogen source and/or amino acids. To test this, we generated gene deletion mutants in two nonessential subunits of TORC1, *TCO89* and the kinase *TOR1*. In addition, we mutated the GTPase *GTR1*, an upstream activator of TORC1 and a component of EGO complex. As reported previously, vegetative growth was strongly reduced in *gtr1*Δ and *tor1*Δ mutants when treated with rapamycin, and was abolished completely in *tco89*Δ cells (S3C Fig) [27, 30]. Upon inhibition of PKA more than 80 percent of mutant cells (*tco89*Δ, *tor1*Δ, and *gtr1*Δ) completed meiosis compared to approximately 20 percent of control cells (Fig 5D). The ability to undergo meiosis was abolished in *tco89*Δ and *gtr1*Δ cells when treated with PKA inhibitor and rapamycin (Fig 5D). Meiosis was not affected in *tor1*Δ cells treated with rapamycin. This can be explained by the presence of the functionally similar Tor2 kinase in TORC1, which can compensate for the *tor1*Δ [27, 29].

Next, we examined how *IME1* promoter activity was affected by *tco89*Δ, *gtr1*Δ, or *tor1*Δ mutations. In cells treated with PKA inhibitor, LacZ activity was significantly higher in all three mutants compared to the control (6h after treatment) (Fig 5E). Moreover, the kinetics and levels of *IME1* induction in the mutant cells treated with PKA inhibitor alone closely resembled that of control cells treated with both PKA inhibitor and rapamycin. The *tco89*Δ and *gtr1*Δ cells treated with rapamycin and PKA inhibitors did not express *IME1*, which is consistent with the observation that these mutants did not induce meiosis under this condition. As expected, in *tor1*Δ mutant cells, rapamycin only had a minor effect on *IME1* promoter activity.

In order to compare the *tco89*Δ mutant to control cells more closely, we monitored *IME1* induction and meiosis in a detailed time-course. When PKA was inhibited in *tco89*Δ cells, *IME1* promoter activity increased significantly faster than in control cells (Fig 5F). In contrast, the *IME1* promoter was not induced even at later time points (12h or 24h) in *tco89*Δ cells treated with rapamycin and PKA inhibitors. Moreover, the kinetics of meiosis in *tco89*Δ cells treated with PKA inhibitor alone closely resembled that of control cells treated with PKA and TORC1 inhibitors and both underwent meiosis efficiently (Fig 5G). These data show that a certain level TORC1 activity is required for *IME1* transcription and entry into sporulation. When TORC1 activity is high or completely blocked, *IME1* expression and sporulation are repressed.

### Inactive TORC1 represses *IME1* mRNA expression

**Fig 6:**
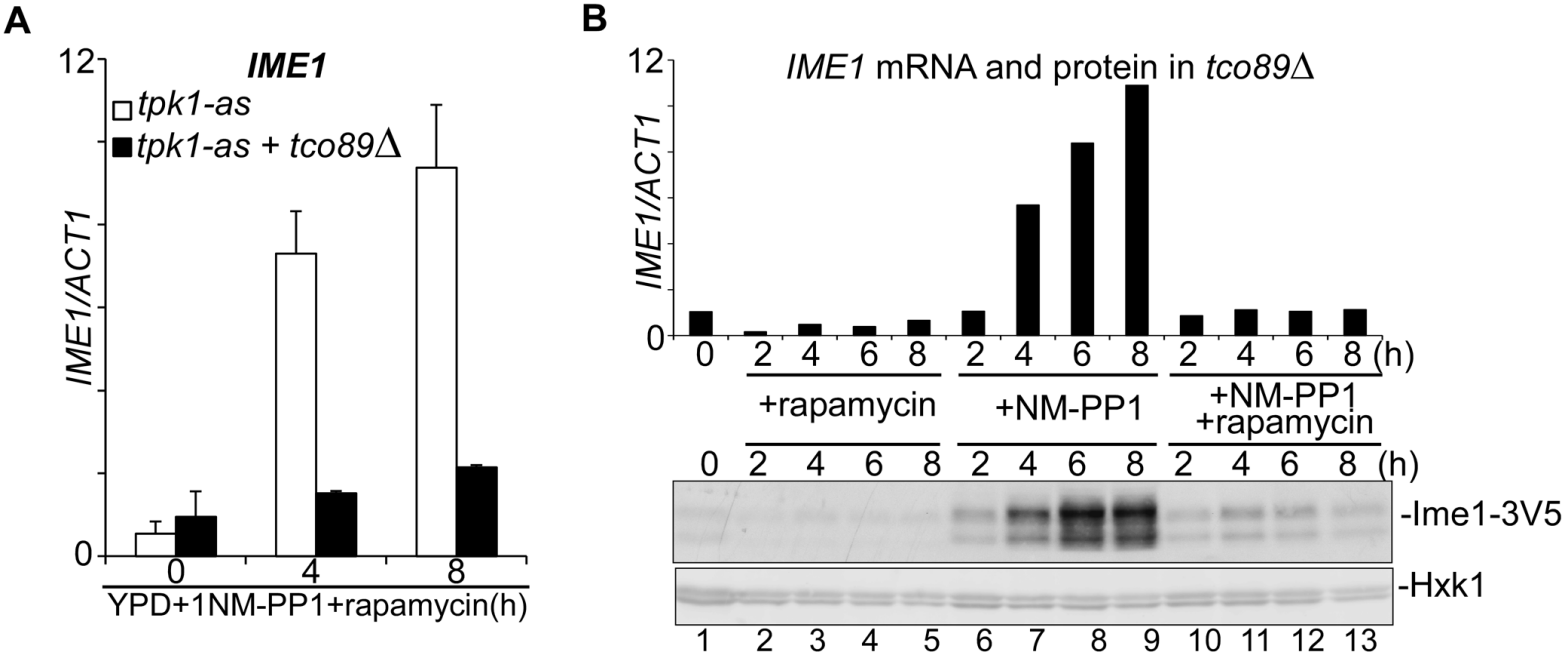
Inactive TORC1 represses *IME1*. (A) *IME1* mRNA quantification in control (FW1976) and *tco89*Δ (FW2154) cells shifted from YPD to YPD plus rapamycin and 1NM-PP1. Samples were taken at the indicated time points. Total RNA was isolated, reverse transcribed, and *IME1* mRNA levels were measured by quantitative PCR. Signals were normalized to *ACT1* levels. The standard error of the mean of at least two biological experiments is shown. (B) *IME1* mRNA and Ime1 protein quantification in *tco89*Δ cells expressing Ime1-V5 (FW2403). Cells were grown in YPD overnight, diluted into YPD and treated with rapamycin, 1NM-PP1, or both compounds. Samples were taken at the indicated time points, and *IME1* mRNA levels were measured as described in A. Ime1 protein levels were quantified by western blot with antibodies direct against V5 and Hxk1 (control).

Several mechanisms could be responsible for the observation that complete inactivation of TORC1 prevents *pIME1-LacZ* activity. Given that TORC1 regulates translation and ribosome biogenesis, one plausible explanation is that the β-galactosidase protein is not produced [11]. Another possibility is that mRNA production or stability is affected by inactive TORC1. To distinguish between these two possibilities, we measured *IME1* mRNA and protein levels in *tco89*Δ cells, rather than *IME1* promoter activity. We found that both *IME1* mRNA and protein levels were strongly reduced, but not completely abolished, in *tco89*Δ cells treated with rapamycin (Fig 6A and 6B, compare lanes 6–9 to 10–13). Furthermore, we observed a small increase in Ime1 protein that correlated with *IME1* mRNA levels following 4 hours of treatment. We conclude *IME1* mRNA accumulation is predominantly affected when TORC1 is completely inhibited.

### TORC1 regulation of *IME1* is mediated by Sch9 kinase

The TORC1 complex has multiple effectors that regulate cellular processes such as autophagy, nitrogen and amino acid sensing, as well as ribosome biogenesis [11]. We investigated whether the Sch9 branch of TORC1 is important for *IME1* regulation.

**Fig 7:**
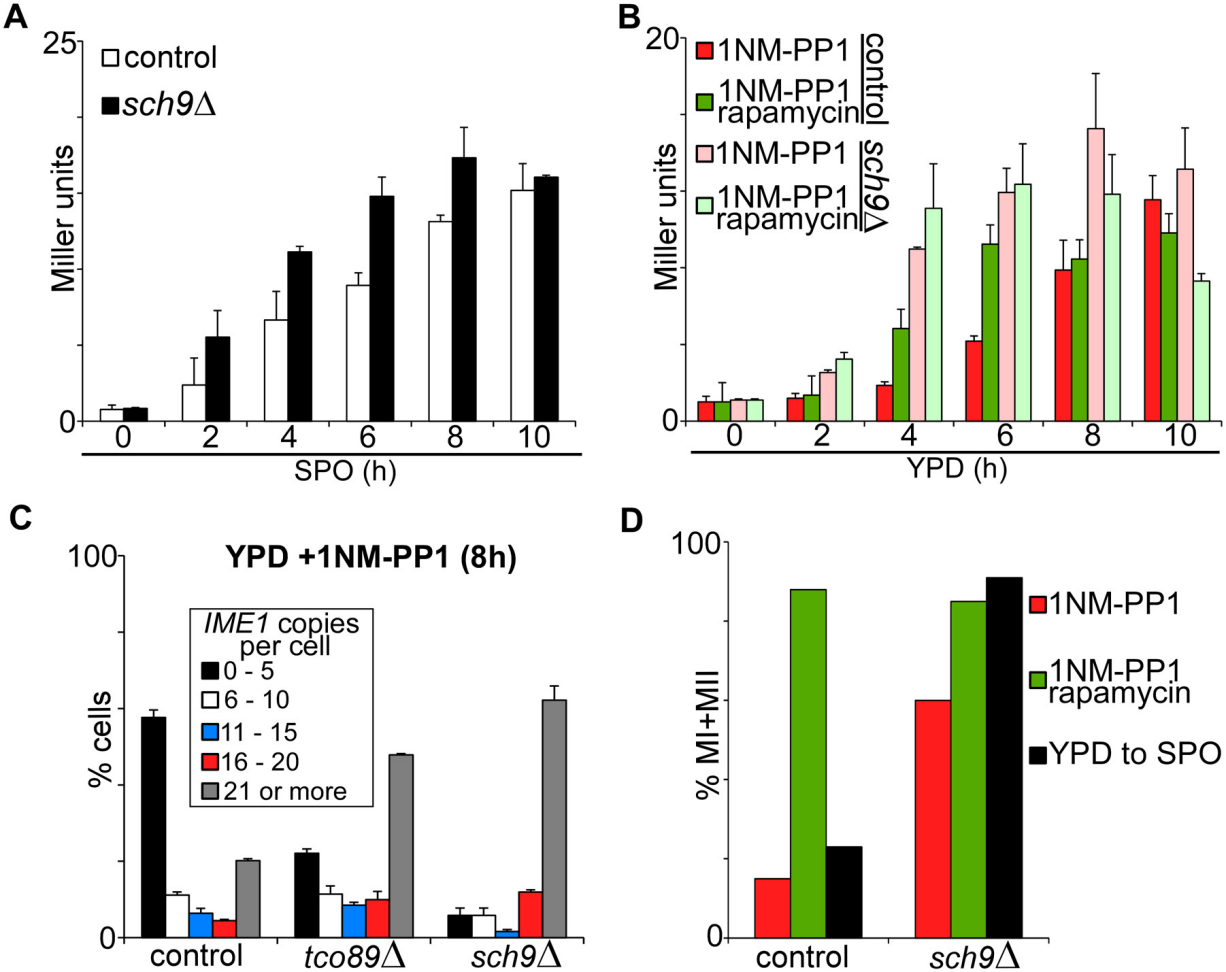
Sch9 controls *IME1* and entry into sporulation. (A) *IME1* promoter activity was measured in strains harbouring *tpk1-as* and *pIME1-LacZ* (control, FW1976) and *sch9*Δ (FW2498). Cells were grown in YPD overnight shifted to SPO and samples were taken at the indicated time points. β-galactosidase activity was measured using a quantitative liquid ortho-Nitrophenyl-β-galactoside (ONPG) assay (see Materials and Methods for details). The promoter activities are displayed in Miller Units, and the standard error of the mean of at least two biological experiments is shown. (B) Similar to A except that cells were diluted into YPD with 1NM-PP1 or 1NM-PP1 plus rapamycin. (C) *IME1* transcript distribution among single cells in control (FW1762), *tco89*Δ (FW1966) and *sch9*Δ (FW2437) strains (all in *tpk1-as* background). After 8 hours treatment with 1NM-PP1, cells were fixed, hybridized with probes directed against *IME1* (AF594) and *ACT1* (Cy5). Finally, cells were imaged and transcript levels were quantified (see Materials and Methods for details). *ACT1* was used as an internal control and only cells positive for *ACT1* were selected for the analysis. At least 120 cells (n = 120) were quantified. The error bars represent the standard error of the mean of two biological experiments. (D) Same treatments and strains as A and B, but here the percentage of cells that underwent meiotic divisions (MI+MII) was determined after 48 hours of treatment.

Sch9 is a serine/threonine kinase that controls ribosome biogenesis, autophagy, and entry into stationary phase [31–33]. It is also directly phosphorylated by Tor1 [34]. First, we quantified *IME1* promoter activity in *sch9*Δ mutant cells shifted from YPD to SPO medium. We found that *IME1* promoter activity was only slightly higher in *sch9*Δ cells compared to control cells (Fig 7A). We next measured *IME1* promoter activity in cells grown in rich medium using the *tpk1-as* allele. Upon inhibition of PKA, *IME1* promoter activity was overall higher and accumulated with faster kinetics in *sch9*Δ cells compared to control cells (Fig 7B). These data indicate that Sch9 is a repressor of *IME1*. If Sch9 is the only downstream target of TORC1 that represses *IME1* than lowering TORC1 activity in *sch9*Δ cells should not further affect *IME1* expression. Indeed, rapamycin treatment did not further increase *IME1* promoter activity in *sch9*Δ cells (Fig 7B, compare pink bars with light green bars). These results indicate that Sch9 mediates repression of *IME1* by TORC1.

To further analyse how Sch9 controls sporulation we measured *IME1* levels in single cells by smFISH. We observed that in *sch9*Δ cells treated with PKA inhibitors the majority (more than 60%) of cells expressed more than 21 copies per cell (Fig 7C). Only a small fraction of cells did not induce *IME1*. Furthermore, the *IME1* mRNA distribution pattern in *sch9*Δ cells closely resembled that of *tco89*Δ cells, which supports our finding that TORC1 repression of *IME1* is mediated by Sch9. Finally, we measured how meiosis is affected in *sch9*Δ cells. In line with the *IME1* expression data, the percentage of cells that completed meiosis was significantly higher (60% versus less than 20% in the control) in *sch9*Δ cells treated with PKA inhibitor (Fig 7D). In addition, in *sch9*Δ cells shifted from YPD to SPO the percentage of cells that underwent meiosis was substantially higher than in wild-type cells induced to sporulate under these conditions (90% versus 20%). Given that we only observed a small increase in *IME1* under this conditions (Fig 7A), the result suggests that Sch9 may also inhibit meiotic progression downstream of *IME1* induction and regulate events such as *IME2* and *NTD80* induction. In conclusion, TORC1-Sch9 signalling contributes to repressing *IME1* expression and entry in sporulation in nutrients-rich conditions.

### Tup1 mediates TORC1 and PKA control of *IME1* expression

**Fig 8:**
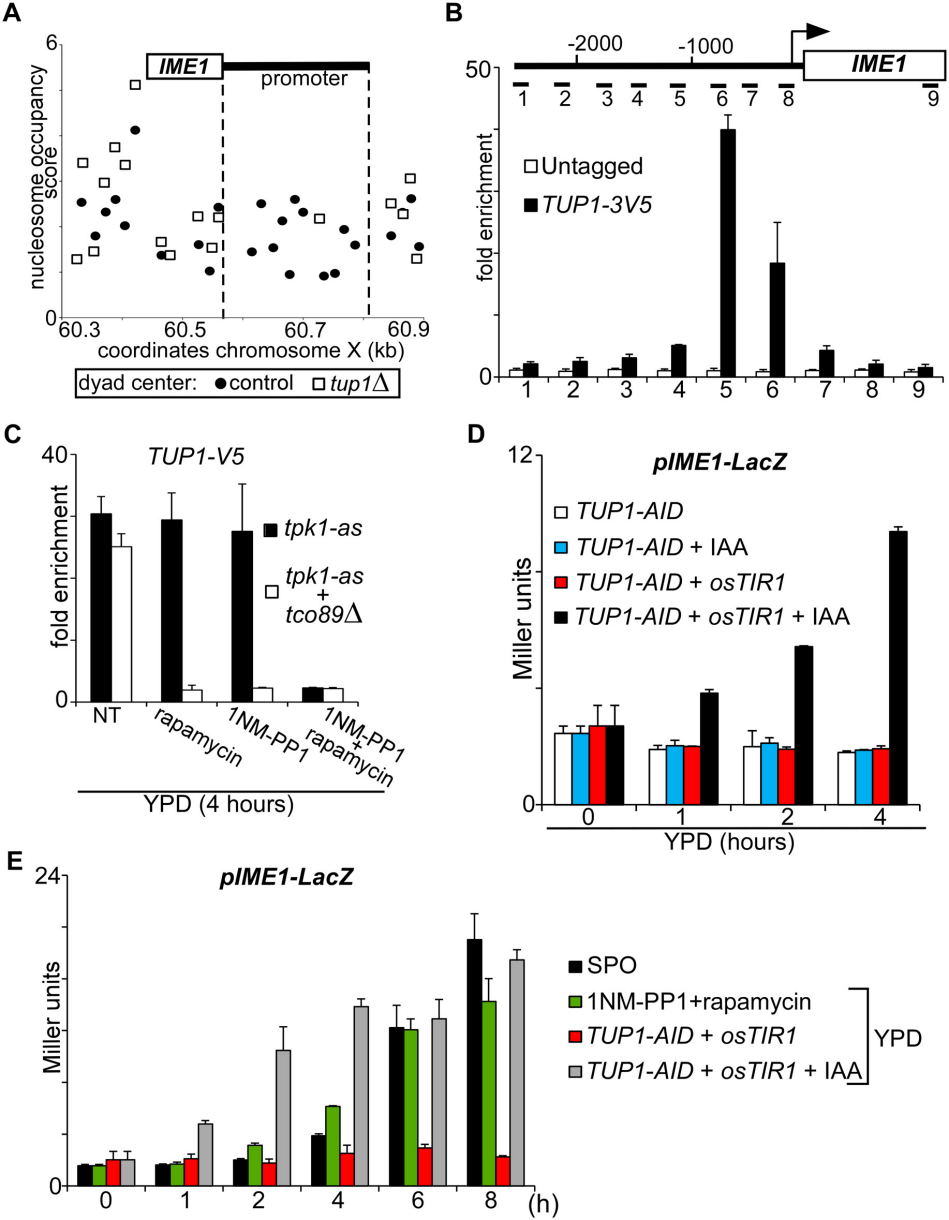
Tup1 binds, represses, and mediates nutrient control of the *IME1* promoter. (A) Data taken from *Rizzo et al*. [39] showing the nucleosome distribution at the *IME1* locus in control (closed circles) and *tup1*Δ mutant (open squares) cells. The x-axis shows the coordinates of the *IME1* locus at chromosome X in kilobases (kb), and y-axis shows the nucleosome occupancy score as described in [39]. The position of each dot or point on the graph represents the coordinate of the nucleosome dyad center at the *IME1* locus. Regions lacking dots are depleted for nucleosomes. (B) Binding of Tup1 to the *IME1* promoter measured by chromatin immunoprecipitation. Diploid cells harbouring *tpk1-as* (control, FW1762) and *tpk1-as* plus Tup1 tagged at the C-terminus with 3xV5 (FW3078) were grown in rich medium (YPD) to mid-log and cross-linked with formaldehyde. Tup1 was immunoprecipitated from chromatin extracts. The recovered DNA was quantified by real-time PCR with 9 different primer sets across the *IME1* promoter and gene. Signals were normalized to the silent mating type locus (*HMR*), which does not bind Tup1. The error bars represent the standard error of the mean of two biological experiments. (C) Tup1 binding to the *IME1* promoter was measured by chromatin immunoprecipitation in control (FW3078) and *tco89*Δ (FW3096) cells. Cells were grown in YPD and shifted to YPD and were either untreated or treated with rapamycin, 1NM-PP1 or both compounds. Tup1 tagged with 3xV5 epitope was immunoprecipitated from chromatin extracts. The recovered DNA was quantified by real-time PCR with primer set five corresponding to middle of the *IME1* promoter. Signals were normalized to the silent mating type locus (*HMR*), which does not bind Tup1. The error bars represent the standard error of the mean of two biological experiments. (D) *IME1* promoter activity upon depletion of Tup1. Cells harbouring *IME1* promoter fused to LacZ (*pIME1-LacZ*) and expressing either Tup1 fused to the auxin induced degron (*TUP1-AID*) (FW3188) or *TUP1-AID* together with *pTEF1-osTIR1* (FW3184) were grown in YPD overnight. Cells were diluted to fresh YPD, either untreated or treated with indole-3-acetic acid (*IAA*) (500 μM), and samples were taken at the indicated time points. β-galactosidase activity was measured using a quantitative liquid ortho-Nitrophenyl-β-galactoside (ONPG) assay (see Materials and Methods for details). The promoter activities are displayed in Miller Units, and the standard error of the mean of at least two biological experiments is shown. (E) Comparison of *IME1* promoter activity during different treatments and growth conditions. Diploid cells harbouring *tpk1-as* and *pIME1-LacZ* (FW1976) were grown overnight in YPD, and diluted to YPD with 1NM-PP1 and rapamycin or cells were washes with water before transferred to sporulation medium. Diploid cells harbouring *TUP1-AID* and *pTEF1-osTIR1* (FW3188) were grown and treated as described D. Samples were taken at the indicated time points, and β-galactosidase activity was measured as described in D.

Having established that PKA and TORC1 control *IME1* expression, we next determined how both signalling pathways control the association of transcription factors with the *IME1* promoter. Several regulators of *IME1* have been identified that regulate *IME1* including the Tup1-Cyc8 complex [5, 35]. This transcriptional repressor is recruited to promoters by sequence specific transcription factors and represses transcription by masking and inhibiting the transcriptional activation domains of transcription factors at gene promoters [36–38]. Several lines of evidence indicate that Tup1 contributes to *IME1* repression. *TUP1* and *CYC8* mutants have been identified in a screen for genes that repress *IME1* expression [35]. In addition, when we plotted the nucleosome occupancy at the *IME1* locus using data from a published genome-wide study, we found that in *tup1*Δ mutant cells the *IME1* promoter is almost completely depleted for nucleosomes suggesting that the promoter is de-repressed (Fig 8A) [39]. Finally, ChIP sequencing data indicated that depletion of Tup1 leads to increased binding of RNA polymerase II to the *IME1* ORF and up-regulation of *IME1* transcription [38].

To examine whether Tup1 directly regulates *IME1*, we measured Tup1 binding across the *IME1* promoter by ChIP in nutrient rich conditions (Fig 8B). We found that Tup1 binds strongly (more than 30 fold over background) to the *IME1* promoter in a region around 800 to 1000 base pairs upstream of the translation start side. To determine whether Tup1 binding to the *IME1* promoter is regulated by nutrients, we treated *tpk1-as* diploid cells with 1NM-PP1 or rapamycin for 4 hours. Tup1 binding to the *IME1* promoter was not affected. However, when we inhibited PKA and TORC1, Tup1 binding to the *IME1* promoter was lost (Fig 8C). Finally, we tested whether the degree of TORC1 activity affected Tup1 binding to the *IME1* promoter. In *tco89*Δ mutant cells Tup1 was bound to the *IME1* promoter in rich medium. Inhibition of PKA in this mutant background was sufficient to disassociate Tup1 from the *IME1* promoter (Fig 8C). Interestingly, when we treated *tco89*Δ cells with rapamycin to inactivate TORC1, Tup1 binding to the *IME1* promoter was also not detectable. We conclude that Tup1 binding to the *IME1* promoter is controlled by PKA and TORC1 activity.

### Depletion of Tup1 leads to rapid activation of *IME1* transcription

We next tested whether Tup1-Cyc8 association with the *IME1* promoter is important for *IME1* repression. We found that Tup1 depletion was sufficient for the activation of the *IME1* promoter (Fig 8D). In diploids cells harbouring a *TUP1-AID* fusion and expressing *pTEF1-osTIR1*, *IME1* promoter activity (*pIME1-LacZ*) increased after treatment with IAA. In contrast, β-galactosidase expression was not induced in untreated cells or cells that only expressed Tup1-AID. Finally, we compared the level of *IME1* induction between Tup1 depleted cells, wild type starved cells (SPO medium), and cells treated with PKA and TORC1 inhibitors grown in rich medium (Fig 8E). Overall *IME1* promoter activity was similar under the different conditions, but increased more rapidly in Tup1 depleted cells compared to cells starved in SPO medium or treated with PKA and TORC1 inhibitors. We conclude that Tup1-Cyc8 is a key repressor of the *IME1* promoter, and that PKA and TORC1 control Tup1 association with the *IME1* promoter.

## Discussion

In yeast, the decision to stop vegetative growth and enter gametogenesis is dictated by nutrient availability regulating the expression of the master regulator *IME1*. Here, we describe how nutrient sensing and signalling regulate *IME1* expression.

### Entry into sporulation requires cooperation between PKA and TORC1 signalling

Previous work implicated both the PKA and TORC1 signalling pathways in regulating *IME1*. Constitutively active PKA, as observed in hyperactivated *RAS2* and in *BCY1* loss of function mutants, inhibits sporulation [9, 10]. Conversely, when PKA signalling is inhibited or reduced, sporulation occurs in a subpopulation of cells even in nutrient-rich conditions. Furthermore, inhibition of TORC1 with rapamycin leads to *IME1* induction and sporulation in saturated YPD cultures [7, 21]. Our work shows that entry into sporulation can be achieved in nutrient-rich conditions by inhibiting PKA and lowering TORC1 signalling. Inhibition of these pathways leads to sporulation with similar kinetics and efficiency as starvation induced sporulation. It has been shown that multiple other signalling pathways can also contribute to *IME1* regulation including G1 cyclins, several MAPK pathways and the Snf1 pathway [40–45]. Given that PKA and TORC1 signalling control the phosphorylation status of a large number of proteins [46, 47], we propose that some of the previously described regulators of *IME1* act downstream of PKA and TORC1 signalling. Further work is needed to decipher how the different signalling networks are connected to each other and how they control entry into sporulation.

Our data suggests that TORC1 and PKA do not only control *IME1*, but also downstream events such as meiotic divisions and packaging into spores. For example, in cells with low PKA, inhibition of TORC1 with rapamycin further stimulates *IME1* induction but also has a profound effect on progression into meiotic divisions and spore formation (Fig 3A). How PKA and TORC1 control other stages of sporulation is not well understood. Our observation that triad formation is significantly enhanced and spore viability is reduced when PKA and TORC1 are inhibited implicates that the two pathways must be tightly regulated during gametogenesis. Further analyses is needed to dissect how PKA and TORC1 themselves are controlled throughout sporulation.

### How is repression of *IME1* by TORC1 and PKA mediated?

In our efforts to understand how TORC1 and PKA repress *IME1*, we identified two factors: Sch9 and Tup1. We find that Sch9, a major mediator of TORC1 signalling, negatively regulates *IME1*. Interestingly, Sch9 and PKA are genetically redundant and functionally overlap [48]. Global gene expression analyses indicate that Sch9 and PKA regulate a common set of genes [49]. These observations suggest that PKA and Sch9 may share one or multiple downstream effectors to control *IME1* and entry into sporulation. Indeed, it is known that Sch9 and PKA phosphorylation inhibits the protein kinase Rim15, which is required for quiescence, *IME1* expression and sporulation [32, 50]. However, a constitutive active allele of *RIM15* cannot de-repress *IME1* in the presence of ample nutrients suggesting that Rim15 is not the only target of Sch9 and PKA [51]. PKA and TORC1 could also repress *IME1* expression by controlling G1 cyclins. It was previously shown that the G1 cyclins *CLN1*, *2* and *3* repress *IME1* [40]. Given that TORC1 and PKA control *CLN1-3* expression, it is possible that *CLN1-3* partially mediate PKA and TORC1 repression of *IME1* [52–54]. PKA is also known to phosphorylate the transcription factors Sok2, Msn2/4, Sko1 and Com2, which directly bind and control the *IME1* promoter [15, 42, 55]. Further efforts are needed to identify downstream effectors of PKA and TORC1 that mediate the regulation of *IME1*.

Our data show that the Tup1-Cyc8 complex is a direct repressor of *IME1* that mediates the signals coming from PKA and TORC1. Tup1 binds to the *IME1* promoter in nutrient rich conditions, but dissociates from the promoter when both PKA and TORC1 are inhibited. The Tup1-Cyc8 complex functions as a global repressor of transcription and is recruited to promoters by sequence specific DNA binding proteins [36–38]. Identifying transcription factors that recruit Tup1 to the *IME1* promoter will give important insights into how *IME1* is regulated by TORC1 and PKA signalling. Interestingly, Tup1 depleted cells do not enter sporulation, even though these cells strongly induce *IME1*. It is possible that other downstream factors, which control entry into sporulation, are not activated under these conditions. For example it has been known that Ime1 translation, phosphorylation, and localization are also affected by nutrients [20, 56–58]. In addition, Tup1 is also required for sporulation. Starving Tup1 depleted cells to induce sporulation, did not result in spore formation. We hypothesize that Tup1 also regulates the transcription of genes that are important for preventing sporulation.

### Intermediate levels of TORC1 activity are required for entry into sporulation

**Fig 9:**
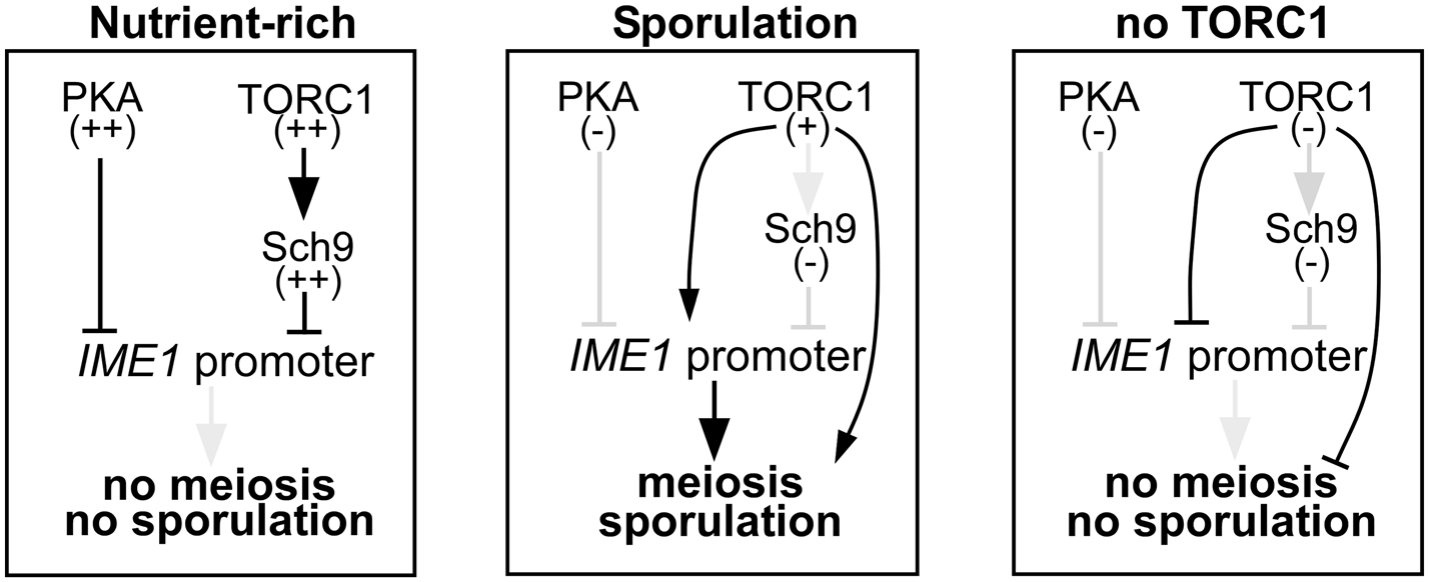
Scheme of how PKA and TORC1 signalling controls *IME1* and entry into sporulation.

Our analyses revealed a positive role for TORC1 in inducing sporulation. When TORC1 is completely inactive, *IME1* is not induced and entry into sporulation does not occur (Figs 5 and 9). We propose that downstream effectors of TORC1 must have opposite effects on *IME1* expression and entry into sporulation. Reduced levels of TORC1 activity are required to inactivate Sch9 (discussed in previous section). Some TORC1 signalling however is needed to induce *IME1* expression via as yet unidentified downstream mediators. Our findings also reconcile two previous contradictory observations regarding the effect of rapamycin on sporulation. Rapamycin treatment was shown to induce *IME1* and sporulation [7, 12, 21] but when rapamycin was combined with nutrient starvation, sporulation was reduced [21]. The observation that intermediate levels of TORC1 are needed for *IME1* induction also implies that there is a defined window of activity to induce sporulation. Given that sporulation is energy consuming, perhaps TORC1 senses whether there are sufficient nutrients available for cells to induce *IME1* and undergo sporulation.

### The effect of ATP/energy production on *IME1*

To facilitate the energy and metabolic needs throughout sporulation, metabolism is finely and dynamically controlled [59, 60]. Mitochondrial respiration activity is essential for both *IME1* expression and sporulation in starvation medium [7, 8]. Previous work showed that inhibition of TORC1 in rich medium induces *IME1* in respiration competent, but not in respiration deficient cells [7]. However, in this study TORC1 activity was inhibited in cells grown to a relatively high density (OD600 = 5.5). It is likely that glucose was already consumed from the medium for some extent and not abundant enough to support *IME1* expression when respiration is blocked. Our system enabled us to challenge the role of respiration during *IME1* induction in rich medium plus glucose. By inhibiting both PKA and TORC1 pathways, we demonstrate that *IME1* can be expressed in respiration deficient cells when a fermentable carbon source such as glucose is available. Thus ATP/energy production via either respiration or fermentation is required for *IME1* activation. It is interesting to speculate that *IME1* functions as an energy sensor that ensures that sporulation is induced by the lack of nutrients and only occurs when the energy source is sufficient for cells to complete sporulation. We further note that the kinetics of *IME1* induction in respiration deficient cells is somewhat slower than in wild-type cells (Fig 4C). Even though we cannot exclude the possibility that other functions of mitochondria are contributing to *IME1* expression, we favour the idea that in wild-type cells glycolysis is simply not sufficient to produce the energy needed for rapid activation of *IME1* expression due to a reduced glucose uptake from the medium. Taken together, we propose that respiration is an essential provider of ATP during starvation induced sporulation.

### Concluding remarks

Signal sensing and signal integration are key determinants of cell fate specification and development. In mammalian cells multiple signals often integrate at master regulatory genes to control cell specialization. The *IME1* promoter serves as a model for signal integration at complex promoters because it can sense multiple nutrient signals and is regulated by transcription of long noncoding RNAs. Understanding the regulation of yeast entry into gametogenesis may shed light on how complex cell fate choices are made in mammalian cells during development.

## Materials and Methods

### Strains and plasmids

SK1 strain background was used for the experiments throughout this manuscript and the genotypes are listed in S2 Table. Gene deletions were generated using one-step deletion protocol as described in [61]. The *tpk1-as* allele was realized by deleting *tpk2*, *tpk3*, and by making a point mutation in *tpk1M164G* as described in [14, 33]. The C-terminal auxin induced degron (AID) tag (used for the *KOG1-AID* and *TUP1-AID* alleles), which also includes three copies of the V5 epitope, was generated using one step PCR integration (the plasmid was a gift from Leon Chan, UC Berkeley). A plasmid expressing *Oryza sativa TIR1* (*osTIR1*) ubiquitin E3 ligase from the *TEF1* promoter was integrated at the *HIS3* locus by digestion with Pme1 (the *pTEF1-osTIR1* plasmid was a gift from Leon Chan, UC Berkeley). Indole-3-acetic acid (IAA) was used to induce depletion of Kog1-AID and Tup1-AID [26].

### Growth conditions

In general, cells were grown overnight in YPD to saturation (1% yeast extract, 2% peptone, 2% glucose) at 30°C, then diluted to fresh YPD (OD600 = 1) and treated with different drugs or shifted to sporulation medium (SPO, 0.3% potassium acetate, 0.002% raffinose, pH 7.0). In some experiments cells were grown overnight in YPD, diluted to pre-sporulation medium (BYTA, 1% yeast extract, 2% tryptone, 1% potassium acetate, 50 mM potassium phtlhalate) for 16 hours, and subsequently shifted to YPD or SPO [62]. Rapamycin was added to cells in a final concentration of 1000 ng/μl unless written otherwise. 1NM-PP1 was added to cells in a final concentration of 3 μM.

### Northern blot analysis

The northern blot protocol for *IME1* was described previously [5].

### RT-PCR

The RT-PCR protocol was described previously [5]. In short, total RNA was treated with DNAse and purified. 750 ng of total RNA was used for the reverse transcription reaction, and single stranded cDNA were quantified by real-time PCR using a SYBR green mix (Life Technologies) on a 7500 Fast Real-Time PCR system (Life Technologies). Signals were normalized to *ACT1* transcripts levels. The primer sequences used are included in S3 Table.

### Western blot analysis and antibodies

A tricarboxylic acid (TCA) extraction protocol was used to make total protein extracts. Samples were separated by SDS page, blotted onto PVDF blotting membrane, and subsequently incubated with anti V5/1:2000 dilution (Life Technologies) and anti-hexokinase/1:8000 dilution (Stratech Scientific) antibodies. As secondary antibodies IRDye800CV/1:15000 dilution (anti-mouse, LI-COR Biosciences) and IRDye680RD/1:15000 dilution (anti-rabbit, LI-COR Biosciences) were used. Western blot images generated using the Odyssey system (LI-COR Biosciences).

### Nuclei/DAPI counting

Cells were fixed overnight in 80% ethanol, and stained with 0.05 μg/ml 4′,6-diamidino-2-phenylindole (DAPI) solution in 100 mM phosphate buffer (pH 7). The number of DAPI masses in at least one hundred cells (n = 100) was counted.

### β-galactosidase liquid assay

Liquid ortho-Nitrophenyl-β-galactoside (ONPG) assay was performed as described previously [5]. In short, 2 ml of OD600 = 1 cell pellets were washed with buffer Z (Phopsphate buffer pH 7, KCl 10 mM, MgCl 1mM) and were snap frozen in liquid nitrogen. Samples for each biological replicate were collected on different days, but ONGP assays were performed together at the same time. Cells were chemically disrupted using Y-PER buffer (Thermo Scientific). Subsequently cells were incubated with ONPG (Sigma) (1 mg/ml in Z buffer plus 50 mM β-mercaptoethanol) till yellow colouring occurred. The reaction was quenches using sodium carbonate (1 mM) and cell debris was cleared by centrifugation. Absorption of each sample was measured at OD420 using a 96 well plate reader. Miller Units were calculated according to a standard formula [63]: Miller Unit = (signal from plate reader (OD420) x 1000) / (cell density (OD600) x time of incubation with ONPG (min)). The data from the experiments represents the standard error of the mean of at least two biological experiments.

### Chromatin immunoprecipitation

Chromatin immunoprecipitation (ChIP) experiments were performed as described previously [5]. Cells were fixed with 1% formaldehyde for 20 min, the reaction was quenched with 125 mM glycine. Cells were disrupted using mini beadbeater (BioSpec), and crosslinked chromatin was sheered by sonication using Bioruptor (Diagenode, 6 cycles of 30 sec on/off). Chromatin extracts were then incubated with anti V5 agarose beads (Sigma) for 2 hours at room temperature, and beads were washed accordingly. To measure Tup1 binding, input and ChIP samples were quantified by real-time PCR using SYBR green mix (Life Technologies) and primers corresponding to the *IME1* promoter on a 7500 Fast Real-Time PCR system (Life Technologies). The mating type locus (HMR) was used as a non-binding negative control. The primer sequences used are included in S3 Table.

### Single molecule RNA FISH

The single molecule RNA fish was performed as described previously [5]. In short, cells were fixed with formaldehyde overnight, treated with zymolyase and further fixed in 80% ethanol. Subsequently cells were hybridized with fluorophore labelled probes directed to *IME1* (AF594) and the internal control *ACT1* (Cy5). Cells were imaged using a 100x oil objective, NA 1.4, on a Nikon TI-E imaging system (Nikon). DIC, DAPI, AF594 (*IME1*), Cy5 (*ACT1*) images were collected every 0.3 micron (20 stacks) using an ORCA-FLASH 4.0 camera (Hamamatsu) and NIS-element software (Nikon). ImageJ software was used to make maximum intensity Z projections of the images [64]. Subsequently, StarSearch software (http://rajlab.seas.upenn.edu/StarSearch/launch.html, Raj laboratory, University of Pennsylvania) was used to determine number transcripts in single cells. Comparable thresholds were used to count the RNA foci in single cells. Only cells positive for the internal control *ACT1* were used for the analysis. At least a total n = 60 cells were counted for each experiment.

## Supporting Information

S1 Fig (A) Cells harbouring the *tpk1M164G*, *tpk2*Δ, *tpk3*Δ alleles (*tpk1-as*, FW1762) were grown in YPD overnight, diluted to 0.2 (OD600), and subsequently cells were treated with 1NM-PP1, rapamycin, both or untreated. Cell density (OD600) was measured over time at the indicated time points. (B) *IME1* promoter activity was measured in a diploid control strain harbouring the *IME1* promoter fused to *LacZ* reporter (*pIME1-LacZ*) (FW612), and a strain harbouring *tpk1-as* and *pIME1-LacZ* (FW1976). Cells were grown in YPD overnight, shifted to sporulation medium (SPO), and samples were taken at the indicated time point. β-galactosidase activity was measured using a quantitative liquid ortho-nitrophenyl-β-galactoside (ONPG) assay (see Materials and Methods for details). The promoter activities are displayed in Miller Units, and the standard error of the mean of at least two biological experiments is shown. (C) Similar as B except that *IME1 tpk1-as* (FW1976) cells were diluted into YPD and treated with rapamycin (1000 ng/ml), 1NM-PP1, or both compounds, or shifted to SPO. Samples were taken after 0, 1, 2, and 4 hours. (D) Similar to C, except that samples shifted to YP-acetate (YPA), YPA plus rapamycin or YPA plus 1NM-PP1.
(PDF)

S2 Fig Single molecule RNA FISH of *IME1* in control and *ime1*Δ cells. (A) Representative images used for the analyses of *IME1* and *ACT1* transcript levels in diploid control (FW1511) and *ime1*Δ (FW81) cells. Cells were grown overnight in YPD and shifted to sporulation medium for 3 hours. Cells were fixed, hybridized with probes directed against *IME1* (AF594) and *ACT1* (Cy5), and imaged (see Materials and Methods for details). *ACT1* was used as an internal control and only *ACT1* positive cells were selected for the analysis. (B) Mean of *IME1* and *ACT1* transcripts’ number among single cells as described A. At least, 60 cells (n = 60) were quantified per time point. The standard error of the mean of at least two biological experiments is shown.
(PDF)

S3 Fig Inactive TORC1 represses *IME1*. (A) Spot assay of strains harbouring *KOG1-AID* (FW1894), *Oryza sativa TIR1* (*pTEF1-osTIR1*) (FW1818), and the combined *KOG1-AID*/*pTEF1-osTIR1* in haploid (FW1887) and diploid (FW1905) cells. Cells were grown in YPD overnight and spotted in five-fold serial dilutions on YPD agar plates in the absence or presence of indole-3-acetic acid (*IAA*) (500 μM). (B) Western blot analysis of Kog1-AID in the absence or presence of IAA. *KOG1-AID*/*pTEF1-osTIR1* expressing cells (FW1887) were grown in YPD overnight, diluted into fresh YPD, and treated with IAA. Samples were taken at the indicated time points. Kog1-AID protein levels were quantified by western blot with antibodies directed against V5 and Hxk1 (control). (C) Doubling times of control (FW1976), *tco89*Δ (FW2154), *gtr1*Δ (FW2164) and *tor1*Δ (FW2162) strains. Cells were grown overnight, diluted into fresh YPD in the absence of presence of rapamycin and a growth curve was determined by OD600 readings. Doubling times were calculated from the exponential part of the growth curve.
(PDF)

S1 Table Sporulation efficiencies. Sporulation efficiencies during different conditions as described in Fig 1.
(DOCX)

S2 Table Table of yeast strains. (DOCX)

S3 Table Oligonucleotide sequence information. (DOCX)

S1 Data Supporting data. (XLSX)
