# Supplementary figures and images for: Nutrient Control of Yeast Gametogenesis Is Mediated by TORC1, PKA and Energy Availability
Source: PLoS Genet. 2016 Jun 6;12(6):e1006075. doi: 10.1371/journal.pgen.1006075 (PMC4894626; doi:10.1371/journal.pgen.1006075)

**A**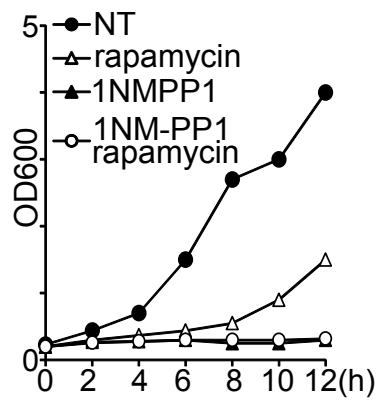**B**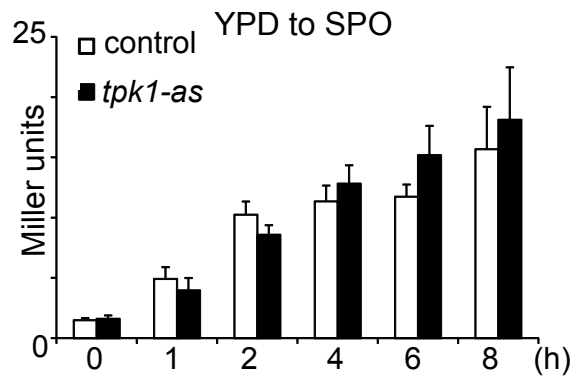**C**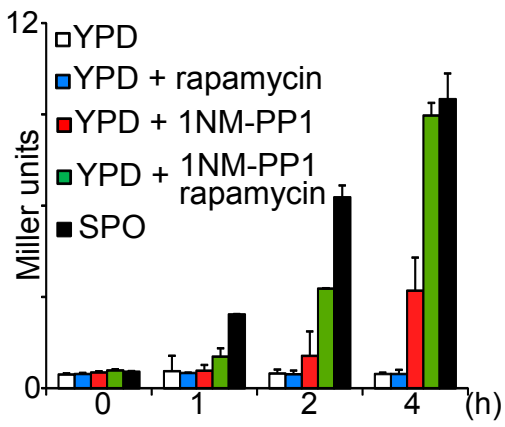**D**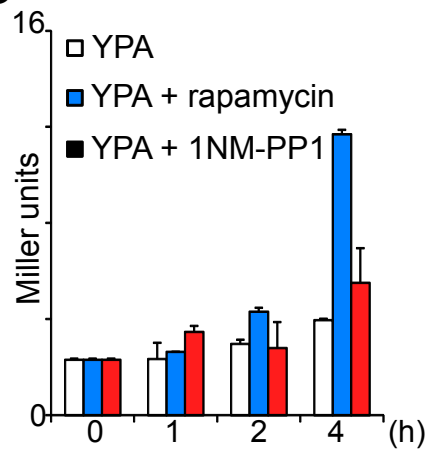

Supplement: S1 Fig — (A) Cells harbouring the tpk1M164G, tpk2Δ, tpk3Δ alleles (tpk1-as, FW1762) were grown in YPD overnight, diluted to 0.2 (OD600), and subsequently cells were treated with 1NM-PP1, rapamycin, both or untreated. Cell density (OD600) was measured over time at the indicated time points. (B) IME1 promoter activity was measured in a diploid control strain harbouring the IME1 promoter fused to LacZ reporter (pIME1-LacZ) (FW612), and a strain harbouring tpk1-as and pIME1-LacZ (FW1976). Cells were grown in YPD overnight, shifted to sporulation medium (SPO), and samples were taken at the indicated time point. β-galactosidase activity was measured using a quantitative liquid ortho-nitrophenyl-β-galactoside (ONPG) assay (see Materials and Methods for details). The promoter activities are displayed in Miller Units, and the standard error of the mean of at least two biological experiments is shown. (C) Similar as B except that IME1 tpk1-as (FW1976) cells were diluted into YPD and treated with rapamycin (1000 ng/ml), 1NM-PP1, or both compounds, or shifted to SPO. Samples were taken after 0, 1, 2, and 4 hours. (D) Similar to C, except that samples shifted to YP-acetate (YPA), YPA plus rapamycin or YPA plus 1NM-PP1. (PDF) [file pgen.1006075.s001.pdf]

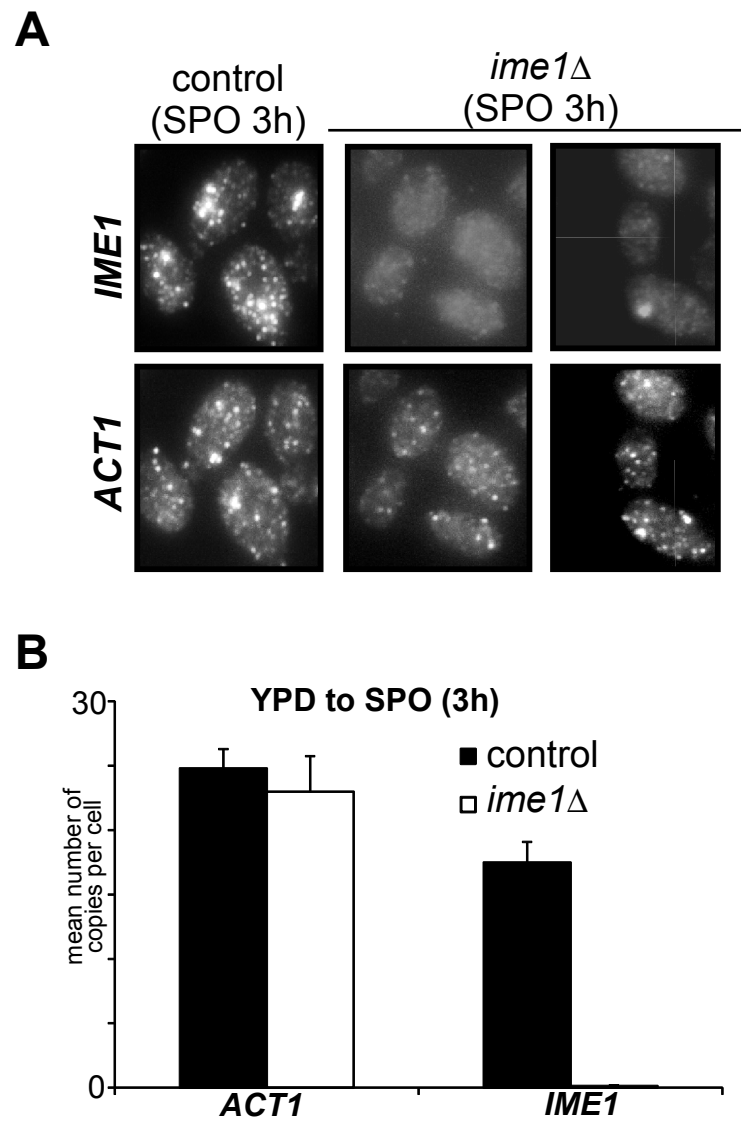

Supplement: S2 Fig — (A) Representative images used for the analyses of IME1 and ACT1 transcript levels in diploid control (FW1511) and ime1Δ (FW81) cells. Cells were grown overnight in YPD and shifted to sporulation medium for 3 hours. Cells were fixed, hybridized with probes directed against IME1 (AF594) and ACT1 (Cy5), and imaged (see Materials and Methods for details). ACT1 was used as an internal control and only ACT1 positive cells were selected for the analysis. (B) Mean of IME1 and ACT1 transcripts’ number among single cells as described A. At least, 60 cells (n = 60) were quantified per time point. The standard error of the mean of at least two biological experiments is shown. (PDF) [file pgen.1006075.s002.pdf]

**A**

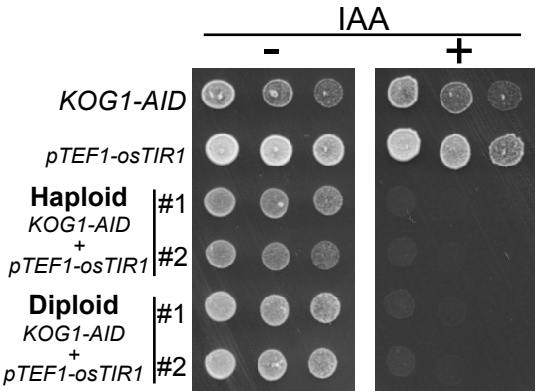

**B**

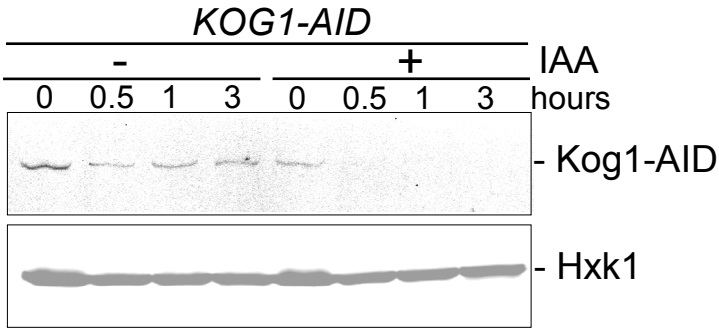

**C**

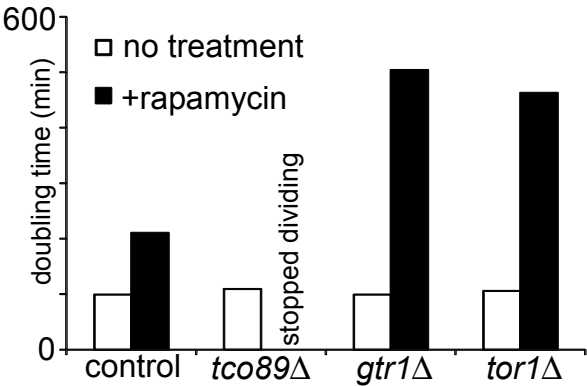

Supplement: S3 Fig — (A) Spot assay of strains harbouring KOG1-AID (FW1894), Oryza sativa TIR1 (pTEF1-osTIR1) (FW1818), and the combined KOG1-AID/pTEF1-osTIR1 in haploid (FW1887) and diploid (FW1905) cells. Cells were grown in YPD overnight and spotted in five-fold serial dilutions on YPD agar plates in the absence or presence of indole-3-acetic acid (IAA) (500 μM). (B) Western blot analysis of Kog1-AID in the absence or presence of IAA. KOG1-AID/pTEF1-osTIR1 expressing cells (FW1887) were grown in YPD overnight, diluted into fresh YPD, and treated with IAA. Samples were taken at the indicated time points. Kog1-AID protein levels were quantified by western blot with antibodies directed against V5 and Hxk1 (control). (C) Doubling times of control (FW1976), tco89Δ (FW2154), gtr1Δ (FW2164) and tor1Δ (FW2162) strains. Cells were grown overnight, diluted into fresh YPD in the absence of presence of rapamycin and a growth curve was determined by OD600 readings. Doubling times were calculated from the exponential part of the growth curve. (PDF) [file pgen.1006075.s003.pdf]
