# Supplementary material for: Nutrient Control of Yeast Gametogenesis Is Mediated by TORC1, PKA and Energy Availability
Source: PLoS Genet. 2016 Jun 6;12(6):e1006075. doi: 10.1371/journal.pgen.1006075 (PMC4894626; doi:10.1371/journal.pgen.1006075)
Supplement: S1 Table — Sporulation efficiencies during different conditions as described in Fig 1. (DOCX) [file pgen.1006075.s004.docx]

**S1 Table. Sporulation efficiencies.** Sporulation efficiencies during different conditions as described in Fig 1.

|  | sporulation after 24 hours | standard error |
| --- | --- | --- |
| YPD | 0.0 | 0.0 |
| YPD to SPO | 47.3 | 4.5 |
| SPO + 2% glucose | 0.0 | 0.0 |
| SPO + 2% glucose + 1NMPP1 | 43.7 | 7.7 |
| YPA | 0.0 | 0.0 |
| YPA+Rapamycin | 94.7 | 0.9 |
| YPA+1NMPP | 64.7 | 6.1 |
| YPD+rapamycin | 0.0 | 0.0 |
| YPD+1NMPP1 | 22.3 | 1.8 |
| YPD+1NMPP1+rapamycin | 84.7 | 3.0 |
|  |  |  |
|  |  |  |
|  |  |  |
