# Supplementary material for: Nutrient Control of Yeast Gametogenesis Is Mediated by TORC1, PKA and Energy Availability
Source: PLoS Genet. 2016 Jun 6;12(6):e1006075. doi: 10.1371/journal.pgen.1006075 (PMC4894626; doi:10.1371/journal.pgen.1006075)
Supplement: S2 Table — (DOCX) [file pgen.1006075.s005.docx]

|  | **S2 Table. Table of yeast strains.** |
| --- | --- |
| FW1511 | *MATa, ho::LYS2, lys2, ura3, leu2::hisG, his3::hisG, trp1::hisG,  MATα, ho::LYS2, lys2, ura3, leu2::hisG, his3::hisG, trp1::hisG,* |
| FW1976 | *MATa, ho::LYS2, lys2, ura3, leu2::hisG, his3::hisG, trp1::hisG, tpk1::tpk1M164G, tpk2::KanMX6, tpk3:: TRP1, IME1::pIME1-LacZ::HIS3  MATα ho::LYS2, lys2, ura3, leu2::hisG, his3::hisG, trp1::hisG, tpk1::tpk1M164G, tpk2::KanMX6, tpk3:: TRP1* |
| FW2437 | *MATa, ho::LYS2, lys2, ura3, leu2::hisG, his3::hisG, trp1::hisG, tpk1::tpk1M164G, tpk2::KanMX6, tpk3:: TRP1, sch9::NatMX*  *MATα ho::LYS2, lys2, ura3, leu2::hisG, his3::hisG, trp1::hisG, tpk1::tpk1M164G, tpk2::KanMX6, tpk3:: TRP1, sch9::NatMX* |
| FW1966 | *MATa, ho::LYS2, lys2, ura3, leu2::hisG, his3::hisG, trp1::hisG, tpk1::tpk1M164G, tpk2::KanMX6, tpk3:: TRP1, tco89::HIS3MX MATα ho::LYS2, lys2, ura3, leu2::hisG, his3::hisG, trp1::hisG, tpk1::tpk1M164G, tpk2::KanMX6, tpk3:: TRP1, tco89::HIS3MX* |
| FW1770 | *MATa, ho::LYS2, lys2, ura3, leu2::hisG, his3::hisG, trp1::hisG, tpk1::tpk1M164G, tpk2::KanMX6, tpk3:: TRP1, pet100::KanMX6  MATα ho::LYS2, lys2, ura3, leu2::hisG, his3::hisG, trp1::hisG, tpk1::tpk1M164G, tpk2::KanMX6, tpk3:: TRP1, pet100::KanMX6* |
| FW2154 | *MATa, ho::LYS2, lys2, ura3, leu2::hisG, his3::hisG, trp1::hisG, tpk1::tpk1M164G, tpk2::KanMX6, tpk3:: TRP1, tco89::HIS3MX, IME1::pIME1-LacZ::HIS3  MATα ho::LYS2, lys2, ura3, leu2::hisG, his3::hisG, trp1::hisG, tpk1::tpk1M164G, tpk2::KanMX6, tpk3:: TRP1, tco89::HIS3MX* |
| FW2164 | *MATa, ho::LYS2, lys2, ura3, leu2::hisG, his3::hisG, trp1::hisG, tpk1::tpk1M164G, tpk2::KanMX6, tpk3:: TRP1, gtr1::NatMX, IME1::pIME1-LacZ::HIS3  MATα ho::LYS2, lys2, ura3, leu2::hisG, his3::hisG, trp1::hisG, tpk1::tpk1M164G, tpk2::KanMX6, tpk3:: TRP1, gtr1::NatMX* |
| FW2162 | *MATa, ho::LYS2, lys2, ura3, leu2::hisG, his3::hisG, trp1::hisG, tpk1::tpk1M164G, tpk2::KanMX6, tpk3:: TRP1, tor1::NatMX, IME1::pIME1-LacZ::HIS3  MATα ho::LYS2, lys2, ura3, leu2::hisG, his3::hisG, trp1::hisG, tpk1::tpk1M164G, tpk2::KanMX6, tpk3:: TRP1, tor1::NatMX* |
| FW2403 | *MATa, ho::LYS2, lys2, ura3, leu2::hisG, his3::hisG, trp1::hisG, tpk1::tpk1M164G, tpk2::KanMX6, tpk3:: TRP1, tco89::HIS3MX, ime1::IME1-3xV5::HIS3 MATα ho::LYS2, lys2, ura3, leu2::hisG, his3::hisG, trp1::hisG, tpk1::tpk1M164G, tpk2::KanMX6, tpk3:: TRP1, tco89::HIS3MX, ime1::IME1-3xV5::HIS3* |
| FW1762 | *MATa, ho::LYS2, lys2, ura3, leu2::hisG, his3::hisG, trp1::hisG, tpk1::tpk1M164G, tpk2::KanMX6, tpk3:: TRP1 MATα ho::LYS2, lys2, ura3, leu2::hisG, his3::hisG, trp1::hisG, tpk1::tpk1M164G, tpk2::KanMX6, tpk3:: TRP1* |
| FW1894 | *MATa, ho::LYS2, lys2, ura3, leu2::hisG, his3::hisG, trp1::hisG, kog1::KOG1-3xV5-IAA7::KanMX6* |
| FW1818 | *MATa, ho::LYS2, lys2, ura3, leu2::hisG, trp1::hisG, his3::pTEF1-osTIR::HIS3* |
| FW1887 | *MATa, ho::LYS2, lys2, ura3, leu2::hisG, trp1::hisG, his3::pTEF1-osTIR::HIS3, kog1::KOG1-3xV5-IAA7::KanMX6* |
| FW1905 | *MATa, ho::LYS2, lys2, ura3, leu2::hisG, trp1::hisG, his3::pTEF1-osTIR::HIS3, kog1::KOG1-3xV5-IAA7::KanMX6*  *MATα, ho::LYS2, lys2, ura3, leu2::hisG, trp1::hisG, his3::pTEF1-osTIR::HIS3, kog1::KOG1-3xV5-IAA7::KanMX6* |
| FW1904 | *MATa, ho::LYS2, lys2, ura3, leu2::hisG, trp1::hisG, tpk1::tpk1M164G, tpk2::KanMX6, tpk3:: TRP1 his3::pTEF1-osTIR::HIS3, kog1::KOG1-3xV5-IAA7::KanMX6*  *MATα, ho::LYS2, lys2, ura3, leu2::hisG, trp1::hisG, tpk1::tpk1M164G, tpk2::KanMX6, tpk3:: TRP1 his3::pTEF1-osTIR::HIS3, kog1::KOG1-3xV5-IAA7::KanMX6* |
| FW2498 | *MATa, ho::LYS2, lys2, ura3, leu2::hisG, his3::hisG, trp1::hisG, tpk1::tpk1M164G, tpk2::KanMX6, tpk3:: TRP1, sch9::NatMX, IME1::pIME1-LacZ::HIS3  MATα ho::LYS2, lys2, ura3, leu2::hisG, his3::hisG, trp1::hisG, tpk1::tpk1M164G, tpk2::KanMX6, tpk3:: TRP1, sch9::NatMX* |
| FW3184 | *MATa, ho::LYS2, lys2, ura3, leu2::hisG, trp1::hisG, tpk1::tpk1M164G, tpk2::KanMX6, tpk3:: TRP1, his3::pTEF1-osTIR::HIS3, tup1::TUP1-3xV5-IAA7::KanMX6, IME1::pIME1-LacZ::HIS3*  *MATα, ho::LYS2, lys2, ura3, leu2::hisG, trp1::hisG, tpk1::tpk1M164G, tpk2::KanMX6, tpk3:: TRP1 his3::pTEF1-osTIR::HIS3, tup1::TUP1-3xV5-IAA7::KanMX6* |
| FW3188 | *MATa, ho::LYS2, lys2, ura3, leu2::hisG, his3::hisG, trp1::hisG, tpk1::tpk1M164G, tpk2::KanMX6, tpk3:: TRP1, tup1::TUP1-3xV5-IAA7::KanMX6, IME1::pIME1-LacZ::HIS3*  *MATα, ho::LYS2, lys2, ura3, leu2::hisG, his3::hisG, trp1::hisG, tpk1::tpk1M164G, tpk2::KanMX6, tpk3:: TRP1 tup1::TUP1-3xV5-IAA7::KanMX6* |
| FW3078 | *MATa, ho::LYS2, lys2, ura3, leu2::hisG, his3::hisG, trp1::hisG, tpk1::tpk1M164G, tpk2::KanMX6, tpk3:: TRP1, tup1::TUP1-3xV5::HIS3 MATα ho::LYS2, lys2, ura3, leu2::hisG, his3::hisG, trp1::hisG, tpk1::tpk1M164G, tpk2::KanMX6, tpk3:: TRP1, tup1::TUP1-3xV5::HIS3* |
| FW3096 | *MATa, ho::LYS2, lys2, ura3, leu2::hisG, his3::hisG, trp1::hisG, tpk1::tpk1M164G, tpk2::KanMX6, tpk3:: TRP1, tco89::HIS3MX, tup1::TUP1-3xV5::HIS3 MATα ho::LYS2, lys2, ura3, leu2::hisG, his3::hisG, trp1::hisG, tpk1::tpk1M164G, tpk2::KanMX6, tpk3:: TRP1, tco89::HIS3MX, tup1::TUP1-3xV5::HIS3* |
| FW3243 | *MATa, ho::LYS2, lys2, leu2::hisG, his3::hisG, trp1::hisG, ime1::pGAL1-IME1, ura3::pGPD1-GAL4(848).ER::URA3*  *MATα ho::LYS2,lys2, leu2::hisG, his3::hisG, trp1::hisG, ime1::pGAL1-IME1, ura3::pGPD1-GAL4(848).ER::URA3* |
| FW612 | *MATa, ho::LYS2, lys2, ura3, leu2::hisG, his3::hisG, trp1::hisG,  MATα, ho::LYS2, lys2, ura3, leu2::hisG, his3::hisG, trp1::hisG, IME1::pIME1-LacZ::URA3* |
| FW81 | *MATa, ho::LYS2, lys2, ura3, leu2::hisG, his3::hisG, trp1::hisG, ime1::HISMX6 MATα, ho::LYS2, lys2, ura3, leu2::hisG, his3::hisG, trp1::hisG, ime1::HISMX6* |
