# Supplementary material for: Nutrient Control of Yeast Gametogenesis Is Mediated by TORC1, PKA and Energy Availability
Source: PLoS Genet. 2016 Jun 6;12(6):e1006075. doi: 10.1371/journal.pgen.1006075 (PMC4894626; doi:10.1371/journal.pgen.1006075)
Supplement: S3 Table — (DOCX) [file pgen.1006075.s006.docx]

**S3 Table. Oligonucleotide sequence information.**

| **Name** | **oligonucleotide sequence** |  | |
| --- | --- | --- | --- |
| primer 1f/IME1RT_f | caacgcctccgataatgtatatg |  | |
| primer 1r/IME1RT_r | acgtcgaaggcaatttctaatg | |  |
| primer 2f | aattcctactggcacccattact |  | |
| primer 2r | ttttgtttgtggggagagga |  | |
| primer 3f | taaacaacaacaacaacgcaca |  | |
| primer 3r | ggcaaggaacaagatcaaaaac |  | |
| primer 4f | cttcgagggaaaggatcaaag |  | |
| primer 4r | ggctgggggttctgtaattc |  | |
| primer 5f | gggtcttaaatacgcagggaat |  | |
| primer 5r | ggcagttcaaaggcttttctta |  | |
| primer 6f | agaaacgcaaatgctcagagag |  | |
| primer 6r | gaggtaatagcggatgacatcaa |  | |
| primer 7f | gccaacttggagaaagaatgtg |  | |
| primer 7r | cggaggtactagtcatcggaat |  | |
| primer 8f | ccgtatggtgttggagtaatttg |  | |
| primer 8r | tgccatttagtggacttcttgag |  | |
| primer 9f | tttcgcgatgaacaacatct |  | |
| primer 9r | ttttatgccaaccctccatc |  | |
| HMR f | acgatccccgtccaagttatg |  | |
| HMR r | cttcaaaggagtcttaatttccctg |  | |
| ACT1RT f | gtaccaccatgttcccaggtatt |  | |
| ACT1RT r | caagatagaaccaccaatccaga | | |
